# Scaling-Down Mass Ivermectin Treatment for Onchocerciasis Elimination: Modeling the Impact of the Geographical Unit for Decision Making

**DOI:** 10.1093/cid/ciab238

**Published:** 2021-06-14

**Authors:** Wilma A Stolk, David J Blok, Jonathan I D Hamley, Paul T Cantey, Sake J de Vlas, Martin Walker, María-Gloria Basáñez

**Affiliations:** 1 Department of Public Health, Erasmus MC, University Medical Center Rotterdam, Rotterdam, The Netherlands; 2 London Centre for Neglected Tropical Disease Research, Department of Infectious Disease Epidemiology, School of Public Health, Faculty of Medicine (St Mary’s Campus), Imperial College London, London, United Kingdom; 3 MRC Centre for Global Infectious Disease Analysis, Department of Infectious Disease Epidemiology, School of Public Health, Faculty of Medicine (St Mary’s Campus), Imperial College London, London, United Kingdom; 4 Division of Parasitic Diseases and Malaria, US Centers for Disease Control and Prevention, Atlanta, Georgia, USA; 5 London Centre for Neglected Tropical Disease Research, Department of Pathobiology and Population Sciences, Royal Veterinary College, University of London, Hatfield, United Kingdom

**Keywords:** onchocerciasis, elimination, ivermectin, mass drug administration, mathematical modeling

## Abstract

**Background:**

Due to spatial heterogeneity in onchocerciasis transmission, the duration of ivermectin mass drug administration (MDA) required for eliminating onchocerciasis will vary within endemic areas and the occurrence of transmission “hotspots” is inevitable. The geographical scale at which stop-MDA decisions are made will be a key driver in how rapidly national programs can scale down active intervention upon achieving the epidemiological targets for elimination.

**Methods:**

We used 2 onchocerciasis models (EPIONCHO-IBM and ONCHOSIM) to predict the likelihood of achieving elimination by 2030 in Africa, accounting for variation in preintervention endemicity levels and histories of ivermectin treatment. We explore how decision making at contrasting geographical scales (community vs larger scale “project”) changes projections on populations still requiring MDA or transitioning to post-treatment surveillance.

**Results:**

The total population considered grows from 118 million people in 2020 to 136 million in 2030. If stop-MDA decisions are made at project level, the number of people requiring treatment declines from 69–118 million in 2020 to 59–118 million in 2030. If stop-MDA decisions are made at community level, the numbers decline from 23–81 million in 2020 to 15–63 million in 2030. The lower estimates in these prediction intervals are based on ONCHOSIM, the upper limits on EPIONCHO-IBM.

**Conclusions:**

The geographical scale at which stop-MDA decisions are made strongly determines how rapidly national onchocerciasis programs can scale down MDA programs. Stopping in portions of project areas or transmission zones would free up human and economic resources.

Onchocerciasis is a neglected tropical disease (NTD) caused by infection with the filarial nematode *Onchocerca volvulus* and transmitted by *Simulium* blackfly vector species. Adult *O. volvulus* worms live in subcutaneous nodules [1], with an average (reproductive) life expectancy of 10 years [2]. Fertilized female worms produce microfilarial progeny, responsible for most of the infection-related clinical manifestations [3]. Currently, the disease still prevails in sub-Saharan Africa (99% of the cases), Yemen, and the Amazonian focus straddling Venezuela and Brazil [4].

Large-scale, regional interventions have been implemented in the last decades. The Onchocerciasis Control Programme in West Africa (OCP; 1974–2002) covered 11 countries and initially deployed vector control through aerial larviciding of vector breeding sites in main river basins, complemented by annual mass drug administration (MDA) of ivermectin in the early 1990s [5]. The African Programme for Onchocerciasis Control (APOC; 1995–2015) introduced annual MDA in the remaining African endemic countries outside the OCP [6], and the Onchocerciasis Elimination Program for the Americas (OEPA; 1993–present) implemented biannual ivermectin MDA in the 6 endemic countries of Latin America [7]. Because of these programs, the public health problem posed by onchocerciasis has been greatly reduced [8, 9]. The goal of elimination (interruption) of transmission has been achieved in 4 countries in the Americas (Mexico, Guatemala, Colombia, and Ecuador) [10] and several isolated foci in Africa [11]. The World Health Organization (WHO), in its 2021–2030 roadmap on NTDs, has proposed that 8 additional countries may be able to achieve elimination of transmission by 2030 [12].

An important challenge for policy makers and program managers is to define when and where MDA can be stopped and post-treatment surveillance should be initiated. The WHO guidelines for stopping MDA and verifying elimination indicate that stop-MDA decisions should be taken at the endemic focus or transmission zone level [13]. These guidelines define a transmission zone as “a geographical area where transmission of *O. volvulus* occurs by locally breeding vectors and which can be regarded as a natural ecological and epidemiological unit for interventions” [13]. Although this definition is applicable in isolated foci, such as the Abu Hamed focus in Sudan [14] and the Galabat-Metema focus at the border of Sudan and Ethiopia, some challenges remain. In the case of the Galabat-Metema focus, there was the need to delineate a 20-km wide buffer zone between the focus and a neighboring transmission zone in the south [15]. Applying the definition becomes more difficult in areas where multiple transmission zones may be contiguous, as in most endemic countries in west and central Africa. Nigeria has chosen a pragmatic approach, taking the state as the unit for stopping MDA in Plateau and Nasarawa without explicitly considering transmission zones [16].

In general, vector biting rates are inversely related to distance from riverine breeding sites, where simuliid larvae and pupae are found [17], and as biting rates are a predictor of onchocerciasis prevalence and infection intensity in endemic villages [18], it follows that distance from ecologically suitable rivers is a predictor of onchocerciasis endemicity [19]. Although not spatially explicit, transmission models suggest that the number of MDA rounds required to bring onchocerciasis prevalence below the elimination threshold in endemic communities strongly depends on preintervention prevalence [20]. While infection may be eliminated after 10–15 years of treatment in lower endemicity settings (presumably located at larger distances from breeding sites), treatment may have to be continued for many more years in higher endemic settings (usually closer to breeding sites) to break the chain of transmission. Thus, the occurrence of ongoing transmission in so-called hotspots is inevitable. Continuation of MDA in the entire transmission area, until the last hotspots are resolved, would lead to overtreatment of places already free of the parasite, using scarce human and economic resources that could be directed to other health priorities. Stopping too early, however, may eventually lead to infection resurgence or re-introduction.

The geographical scale at which decisions are made regarding stopping MDA will be a key driver of how rapidly national programs can transition from active intervention to post-treatment (and post-elimination) surveillance. In this article, we use transmission dynamics modeling to predict how the number of people requiring ivermectin treatment will decline between 2020 and 2030, comparing 2 contrasting decision-making approaches to stop-MDA—namely, at the level of the implementation unit (“project”), as used by the former APOC program, or at the level of endemic communities (villages).

## METHODS

### Models

We use 2 stochastic individual-based simulation models, EPIONCHO-IBM and ONCHOSIM, to predict how onchocerciasis microfilarial prevalence levels decline during MDA and when MDA can be stopped with minimal risk of resurgence. Detailed descriptions of both models are provided elsewhere [8, 18, 21]. Here, we give a brief characterization of their similarities and differences. The 2 models are used side-by-side to capture uncertainties in the underlying transmission processes that influence the predictions [22].

Both EPIONCHO-IBM and ONCHOSIM simulate onchocerciasis transmission in a closed, dynamic population, typically representing a village. The models follow humans in the simulated population over time, keeping track of the number of adult *O. volvulus* (of each sex and reproductive status) and density of skin microfilariae (mf). The dynamics of infection (from ingested mf to infective L3 larvae) in the vector population are modeled deterministically, with both models assuming density-dependent larval establishment within the (savannah) *Simulium damnosum* vectors.

In both models, the presence of male and female worms is required for the production of mf, assuming a polygamous mating system (a male worm can mate with all females within the same human host). Both models account for age- and sex-dependent exposure of humans to blackfly bites, as well as for individual-level heterogeneity in exposure. Yet, the age- and sex-specific patterns of exposure differ between the models, with EPIONCHO-IBM assuming higher exposure to fly bites of under 5-year-old children, particularly boys, than ONCHOSIM. Furthermore, ONCHOSIM assumes that a constant, low proportion of incoming L3 larvae survives to develop into mature adult worms, whereas EPIONCHO assumes that this proportion increases as transmission intensity (individual exposure to L3 larvae) decreases (eg, after MDA). In addition, ONCHOSIM includes excess mortality due to blindness when a threshold microfilarial load is reached, not yet incorporated into EPIONCHO-IBM. Due to these differences, onchocerciasis transmission is harder to eliminate according to EPIONCHO-IBM, leading to a longer required duration of MDA and lower elimination probabilities [22].

Both models capture the impact of MDA in similar ways, assuming that a given proportion (coverage) of the human population is treated per round (excluding children under 5), while a fixed 5% of the population never takes treatment (systematic nonparticipation). Assumptions regarding the efficacy of ivermectin in treated individuals are also harmonized, with both models assuming that ivermectin exerts a powerful microfilaricidal effect and an irreversible effect on adult worm fecundity, decreasing it by approximately 35% following each treatment round.

A description of the parameter values in each model is provided in Supplementary Tables S1 and S2).

### Geographical Scope of the Analysis

Our analysis focused on APOC’s original target area, excluding the following: (1) untreated low-endemic areas that were not previously prioritized for treatment by APOC and still remain to be delineated by onchocerciasis elimination mapping [23]; (2) areas reporting elimination of the vector and/or parasite [14, 24, 25]; (3) areas where MDA was stopped before 2019 as elimination was deemed to have been achieved [16, 26, 27]; (4) meso- and hyperendemic areas identified recently, which were not covered by APOC; and (5) Liberia, as it is geographically separate from the rest of APOC. We include 123 separate MDA implementation units (henceforth referred to as “projects” following APOC’s terminology) in 15 African countries. See Supplementary Table S3 for a list of all projects considered.

### Data on Baseline Endemicity Distribution and Population Size at Pixel Level

From a previously published geostatistical map of nodule prevalences in APOC countries, we extracted the nodule prevalence estimates with their associated standard error for all pixels (raster cells on the map at 0.9- to 0.93-km resolution) per project [28]. Pixel-level nodule prevalence estimates were converted in microfilarial prevalences using the association published by Coffeng et al [29], as explained below. APOC estimates of the project population size in 2015 were divided by the number of pixels covered by a project to estimate the population density. The median population size per pixel was 28.7 (interquartile range, 11.5–55.0). The population density for 2015–2030 was calculated from country-specific rural population growth rates from reference [30]. Spatial variation in population density was not considered.

### Mass Drug Administration Scenarios and Simulation Approach

Ivermectin MDA start year and frequency (annual or biannual) for each project were based on previously developed APOC treatment scenarios for the period 1995–2013 [31], updated based on subsequent evaluation data (J. H. Remme, personal communication, 2020). As in Kim et al [31], we took as start year the first year in which a project reported at least 60% treatment coverage of total population. We assumed that treatment has continued with the same frequency as recorded for 2013, unless information was available about a switch in frequency (eg, Ethiopia). For any projects that had not started in 2013, we assumed that they started in 2014. Recognizing the inaccuracy of reported coverage data, we assumed that 65% of the total population was treated per round for the entire treatment period for all projects, with 5% systematic nonparticipation. Supplementary Table S3 presents an overview of scenario assumptions per project for the period through 2019.

For each project, we varied the year of the last ivermectin treatment from 2019 to 2030 (11 scenarios). After the last treatment round, the simulated period was extended by another 50 years to assess whether or not infection would eventually be eliminated (ie, microfilarial prevalence = 0%). Elimination probabilities are calculated as the proportion of runs in which elimination is achieved.

Each scenario was run 7500 or more times for both models to predict expected trends in microfilarial prevalence for communities of approximately 400 individuals. Assumptions about local transmission conditions were varied between runs to obtain simulations across a range of baseline (preintervention) microfilarial prevalence (20–85%). Supplementary Figures S1 and S2 summarize the modeling assumptions.

### Analysis of Simulation Results

Although pixels do not directly equate to communities, the frequency distribution of preintervention endemicities at a pixel level gives a reasonable reflection of the distribution of preintervention endemicities at a community level. In our analysis, we therefore treated pixels as communities. We converted the nodule prevalence per pixel into microfilarial prevalence following the method of Coffeng et al [29], to obtain an expected frequency distribution of microfilarial prevalences per project. We then assigned a simulation run to each pixel of a project based on the thus-modeled preintervention microfilarial prevalence and assessed whether elimination of transmission was eventually achieved in that pixel. This was repeated 100 times to account for uncertainty in nodule prevalence estimates and the conversion to microfilarial prevalence. The mean proportion of pixels that had achieved elimination by a certain year is interpreted as the proportion of communities no longer requiring treatment if stop-MDA decisions are made at the community level. The number of people still requiring treatment between 2020 and 2030 was calculated by summing the population of pixels that had not yet achieved elimination. See Supplementary Material Section 4 for more details and Supplementary Figures S3–S5 for an illustrative example.

Based on the mean proportion of pixels achieving elimination per project in a given year, we assessed whether the project-level criteria for stopping MDA were met. This was said to be achieved if at least 90% of pixels in the project no longer required treatment in all 100 iterations (ie, accounting for uncertainty in the preintervention microfilarial prevalence arising from the nodule to microfilarial prevalence conversion).

### Good Modeling Practice

Supplementary Table S4 (PRIME-NTD table) describes our adherence to the 5 principles of the NTD Modelling Consortium on good practice for policy-relevant modeling [32].

## RESULTS

**Figure 1.**
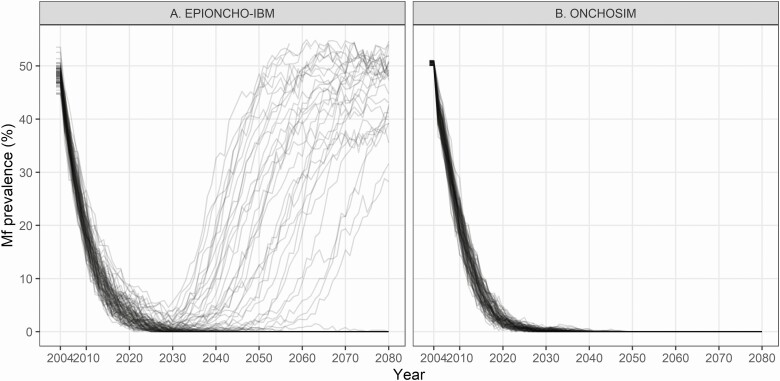
*Onchocerca volvulus* microfilarial (Mf) prevalence trends during and after 26 years of ivermectin MDA (2004–2030) and 50 years after cessation of MDA as predicted by (*A*) EPIONCHO-IBM and (*B*) ONCHOSIM. The black lines represent the trend of individual (stochastic) simulations in settings with a preintervention microfilarial prevalence of 50%. The coverage is assumed to be 65% of the total population with 5% systematic nonparticipation. EPIONCHO-IBM predicts that 63 (66%) of 96 runs lead to elimination, whereas this is 100% for the 100 ONCHOSIM runs. Abbreviation: MDA, mass drug administration.

Figure 1 illustrates the microfilarial prevalence dynamics for settings with a preintervention prevalence of approximately 50% and MDA from 2004 to 2030. Generally, EPIONCHO-IBM and ONCHOSIM both predict a similar prevalence trend during MDA. However, EPIONCHO-IBM predicts more recrudescence than ONCHOSIM after stopping treatment in 2030: recrudescence occured in 34% of all EPIONCHOIBM runs after stopping treatment in 2030, where this happened in none of the ONCHOSIM runs.

**Figure 2.**
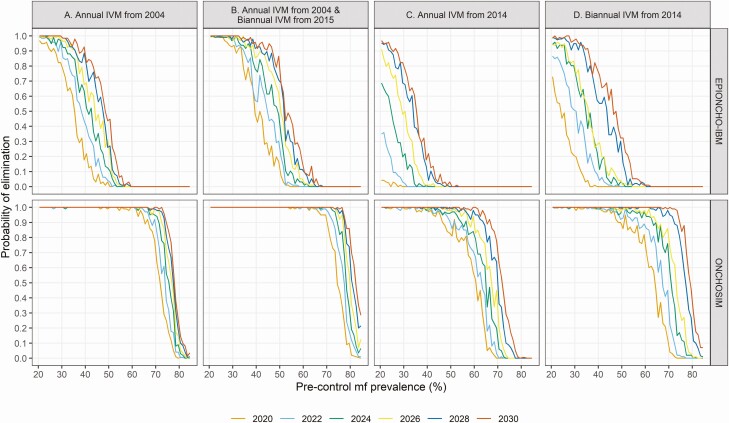
Probability of elimination of transmission at the community (pixel) level by preintervention microfilarial (mf) prevalence as predicted by EPIONCHO-IBM and ONCHOSIM. Graphs from left to right represent different treatment histories: *A*, annual IVM from 2004; *B*, annual IVM from 2004, switching to biannual treatment from 2015 onwards; *C*, annual IVM from 2014; and *D*, biannual IVM from 2014. The stop-year of treatment ranged from 2020 to 2030, represented by the colored lines. The range of preintervention microfilarial prevalence is 20–85%. The coverage is assumed to be 65% of the total population with 5% systematic nonparticipation. Abbreviation: IVM, ivermectin mass drug administration.

The proportion of communities achieving elimination in a given year (2020–2030) depends on baseline endemicity, the start year of MDA (varying in our simulations from 1996 to 2014), and MDA frequency (annual, biannual, switching from annual to biannual). Both models predict a lower probability of elimination if preintervention microfilarial prevalence levels are high (Figure 2). The elimination probability is higher for settings with a long MDA history than for settings with a more recent start of MDA (Figure 2A vs Figure 2C). Switching from annual to biannual MDA or treating biannually from the start of MDA results in higher elimination probabilities than treating annually throughout the program (Figure 2A vs Figure 2B and 2D). Although both models are in agreement qualitatively, EPIONCHO-IBM predicts lower probabilities of elimination.

Due to between-project variation in start year and frequency of MDA, and variation between communities within project areas in preintervention endemicity level, the calendar year in which MDA can safely be stopped varies between and within projects. Supplementary Table S5 provides information by project on the proportion of communities that could stop MDA by 2020, 2025, and 2030, and the year in which treatment would stop in case of project-level decision making.

**Figure 3.**
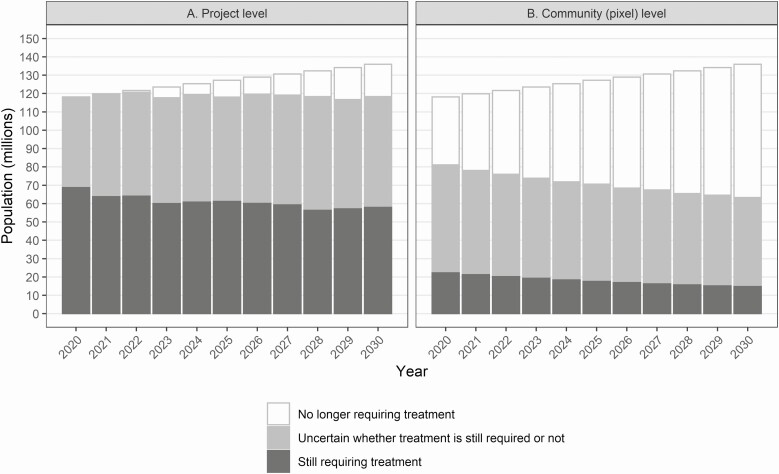
Population requiring ivermectin treatment if stopping-MDA decisions are made at the (*A*) project level and (*B*) community (pixel) level as predicted by EPIONCHO-IBM and ONCHOSIM, aggregated over all projects (*n* = 123). The height of each bar gives the total population size of the 123 projects. The population is subdivided based on predicted numbers requiring treatment. The dark-gray section of each bar indicates the population for which both models agree that treatment is still required. The light-gray section indicates the additional number of people requiring treatment given by EPIONCHO-IBM, whereas ONCHOSIM predicts that treatment is no longer needed in this section. The white section of each bar indicates the population for which both models agree that treatment is no longer required. Abbreviation: MDA, mass drug administration.

Figure 3A shows the trend in the total number of people still requiring treatment across all projects, if stop-MDA decisions are made at project level, accounting for population growth. The deviating model predictions translate into wide prediction intervals, indicated by the light-gray sections of the bars. In particular, ONCHOSIM suggests that treatment is only required in the population indicated by the dark-gray section of each bar, whereas EPIONCHO-IBM suggests that treatment is required in the population indicated by the dark- plus light-gray sections of each bar. Thus, the number of people still requiring treatment was estimated between 69 and 118 million in 2020 and between 59 and 118 million in 2030. As shown in Figure 3B, the population still requiring treatment would be substantially lower if stop-MDA decisions were made at a community level: between 23 and 81 million for 2020 and between 15 and 63 million in 2030.

The same uncertainty affects the converse estimates of the number of people no longer requiring treatment. In case of project-level decision making, these estimates ranged from less than 0.5 to 49 million in 2020 and from 18 to 78 million in 2030 (Figure 3A). In case of community-level decision making, these estimates ranged from 37 to 96 million in 2020 and from 73 to 121 million in 2030. Now, the lower limits are based on EPIONCHO-IBM (indicated by the white section of each bar) and the upper limits on ONCHOSIM (indicated by the white plus gray section of each bar).

## Discussion

Our study illustrates how the geographical scale at which stop-MDA decisions are made drives the decline in the number of people requiring treatment, and hence the transitioning of populations to post-treatment and eventually post-elimination surveillance. Overall, the number of people still requiring treatment would be 40–50 million lower if stop-MDA decision were made at a community level relative to a project level. A similar difference is predicted for 2030.

Estimates of the absolute number of people still requiring ivermectin treatment to achieve onchocerciasis elimination should be interpreted with caution, as the simplified history of control scenarios per project may not accurately represent the actual history of control (eg, due to recent changes in policy to accelerate elimination). Also, we assumed that the same coverage and systematic nonparticipation applied everywhere and throughout, ignoring spatiotemporal variations and possible incidental skipping of treatment rounds. The results for project-level decision making further depend on the chosen criteria for stopping MDA. We assumed that MDA could be stopped at the project level if at least 90% of communities in the project no longer required treatment in all 100 iterations. Taking a higher proportion would obviously imply a longer continuation of MDA and vice versa. Nevertheless, the ranges permit a crude appreciation of the differences obtained if decisions to stop ivermectin MDA were made at contrasting geographical scales.

Our estimates of the numbers of people still requiring or no longer requiring treatment are highly uncertain, due to key unknowns in the processes regulating the transmission of *O. volvulus* in human and vector populations. These uncertainties are largely captured by the 2 onchocerciasis models that substantially differ in their assumptions regarding parasite establishment within humans and patterns of exposure to vector bites, among others, for which independent data are scarce. This uncertainty strongly affects elimination predictions, as discussed in more detail elsewhere [22], resulting in wide uncertainty bands in Figure 3.

In the present analysis, treatment cessation was evaluated at the community and project levels, with projects varying greatly in size. Stopping treatment at the community level would substantially reduce the overall population needing treatment. However, making stopping decisions at this level is neither practical (data would have be collected in every endemic village) nor epidemiologically sound (the long flight range of vectors [33, 34] and the movement of humans between communities would lead to re-introduction of infection if transmission still occurs in neighboring communities). The stop-MDA decision needs to be made at a larger geospatial unit than the community, but we recommend using smaller units than an entire project or a transmission zone where possible, as much efficiency can be gained by using a smaller scale.

Stopping in portions of a transmission zone would be particularly useful if infection has been virtually cleared from most of the region and only still appears in some hotspots. This situation occurred in the Galabat-Metema focus in Ethiopia, where transmission was interrupted everywhere apart from the Wudi Gemzu hotspot [15]. The Ethiopian onchocerciasis control program decided to intensify interventions in the Wudi Gemzu hotspot (by quarterly ivermectin mass treatment) and to stop treatment in the surrounding area. A 20-km-wide buffer zone separated the area where treatment was stopped from other areas with potential ongoing transmission. The benefit of stopping treatment in a large part of the area should outweigh the burden from having to implement extra measures or demonstrating the presence of a buffer zone. Additionally, demonstrating progress would increase support and enthusiasm for achieving the long-term goal of country-wide interruption of onchocerciasis transmission among all stakeholders involved. Advanced geostatistical models (such as for *Loa loa* [35]) could be useful tools to put the strategy outlined above into practice, by allowing the identification of transmission hotspots, areas with likely interrupted transmission, and areas where additional data are required.

In conclusion, the geographical scale at which stop-MDA decisions are made strongly determines how rapidly national onchocerciasis programs can scale down mass ivermectin treatment programs. Stopping earlier would free up human and economic resources that can then be used to accelerate elimination in residual transmission areas or to address other health problems.

## Supplementary Data

Supplementary materials are available at *Clinical Infectious Diseases* online. Consisting of data provided by the authors to benefit the reader, the posted materials are not copyedited and are the sole responsibility of the authors, so questions or comments should be addressed to the corresponding author.

ciab238_suppl_Supplementary-Material

## Notes

***Authors’ contributions.*** W. A. S., D. J. B., J. I. D. H., P. T. C., M. W., and M.-G. B. conceived the study and designed the simulations. D. J. B. and J. I. D. H. performed the simulations. D. J. B. and W. A. S. analyzed the simulation results. All authors contributed to the interpretation of the results. W. A. S. drafted the first version of the manuscript. All authors contributed to the writing and provided critical feedback. All authors have read and approved the manuscript.

***Acknowledgments.*** We are grateful to J. Hans Remme for providing pixel-level data on nodule prevalence and population density per project. We thank Stefano Malvolti and Melissa Malhame from MM Global Health (MMGH) Consulting GmbH for their advice throughout the project.

***Supplement sponsorship.*** This article appears as part of the supplement “Sustainable control of neglected tropical diseases – surveillance and diagnostics”, sponsored by the NTD Modelling Consortium.

***Financial support.*** W. A. S., D. J. B., J. I. D. H., S. J. d. V., M. W., and M.-G. B. gratefully acknowledge funding from the NTD Modelling Consortium by the Bill & Melinda Gates Foundation (grant number OPP1184344). M. G. B. acknowledges joint center funding (grant number MR/R015600/1) from the UK Medical Research Council (MRC) and the UK Department for International Development (DFID) under the MRC/DFID Concordat agreement, which is also part of the European and Developing Countries Clinical Trials Partnership (EDCTP2) program supported by the European Union.

***Potential conflicts of interest.*** W. A. S., D. J. B., J. I. D. H., S. J. d. V., M. W., and M.-G. B. report grants from the Bill & Melinda Gates Foundation during the conduct of the study; J. I. D. H. and M.-G. B. report nonfinancial support from the Medical Research Council and Department for International Development, United Kingdom. P. T. C. has nothing to disclose. All authors have submitted the ICMJE Form for Disclosure of Potential Conflicts of Interest. Conflicts that the editors consider relevant to the content of the manuscript have been disclosed.
