# Supplementary material for: Scaling-Down Mass Ivermectin Treatment for Onchocerciasis Elimination: Modeling the Impact of the Geographical Unit for Decision Making
Source: Clin Infect Dis. 2021 Jun 14;72(Suppl 3):S165–71. doi: 10.1093/cid/ciab238 (PMC8201558; doi:10.1093/cid/ciab238)
Supplement: ciab238_suppl_Supplementary-Material [file ciab238_suppl_supplementary-material.pdf]

Supplementary information to:

Scaling-down of mass ivermectin treatment for onchocerciasis elimination: modelling the impact of the geographical unit for decision making

Wilma A. Stolk[1], David J. Blok[1], Jonathan I.D. Hamley[2,3], Paul Cantey[4], Sake J. de Vlas[1], Martin Walker[2,5], María-Gloria Basáñez[2,3]

Affiliations
[1] Department of Public Health, Erasmus MC, University Medical Center Rotterdam, P.O. Box 2040, 3000 CA Rotterdam, The Netherlands;
[2] London Centre for Neglected Tropical Disease Research (LCNTDR), Department of Infectious Disease Epidemiology, School of Public Health, Faculty of Medicine (St Mary's Campus), Imperial College London, Norfolk Place, London W2 1PG, UK;
[3] MRC Centre for Global Infectious Disease Analysis, Department of Infectious Disease Epidemiology, School of Public Health, Faculty of Medicine (St Mary's campus), Imperial College London, Norfolk Place, London W2 1PG, UK;
[4] Division of Parasitic Diseases and Malaria, U.S. Centers for Disease Control and Prevention, Atlanta, Georgia, U.S.A.;
[5] London Centre for Neglected Tropical Disease Research (LCNTDR), Department of Pathobiology and Population Sciences, Royal Veterinary College, University of London, Hatfield AL9 7TA, UK.

Corresponding authors: Wilma A. Stolk, PhD; Department of Public Health, Erasmus MC, University Medical Center Rotterdam, PO Box 2040, 3000 CA Rotterdam, The Netherlands. Phone: +31 10 704 3730; Email: w.stolk@erasmusmc.nl

Alternate corresponding author: David J. Blok, PhD; Department of Public Health, Erasmus MC, University Medical Center Rotterdam, PO Box 2040, 3000 CA Rotterdam, The Netherlands. Phone: +31 10 704 41 77; Email: d.j.blok.1@erasmusmc.nl

Contents

Section 1. EPIONCHO-IBM: model and parameter values 3

1.1 Model version and availability 3

1.2 Model parameters and their values 3

1.3 Parameters values used to simulate varying pre-control onchocerciasis microfilarial prevalence levels in EPIONCHO-IBM 7

Section 2. ONCHOSIM: model and parameter values 8

2.1 Model version and availability 8

2.2 Model parameters and their values 8

2.3 Parameters combinations used to simulate varying pre-control onchocerciasis microfilarial prevalence levels 11

Section 3. Project list with assumptions on history of control, population size, and population distribution over endemicity categories 12

Section 4. Methods for estimating the population requiring treatment by project 19

Section 5. The Policy-Relevant Items for Reporting Models in Epidemiology of Neglected Tropical Diseases 23

Section 6. Results disaggregated by project 25

References 31

Section 1. EPIONCHO-IBM: model and parameter values

1.1 Model version and availability

Model version: EPIOCNHO-IBM.v1.Dec.19.
Simulation programme and programme code: https://github.com/jonathanhamley

1.2 Model parameters and their values

Table S1. EPIONCHO-IBM parameter quantification (Hamley et al. 2019 [1]).

Parameter or variable	Definition	Value and units	Reference
**Human host demography**			
N_H	Number of human hosts in population	440	[1]
μ_H	Mortality rate of human hosts	0.02 year^{-1}	[2]
a_{max}	Maximum age of human hosts	80 years	[3]
ψ'_S	Probability that a human host is of sex s	$\psi'_F = \psi'_M = 0.5$	[3]
**Exposure to blackfly bites**			
k_E, β_E	Shape and rate parameters of the gamma distribution describing individual human host exposure to blackfly bites	0.3	[1]
$Q = E_M/E_F$	Relative male to female exposure to blackfly bites	1.20	[3]
w	Age-specific change in contact rate with vectors for females	-0.023 year^{-1}	[3]
α_M	Age specific change in contact rate with vectors for males	0.007 year^{-1}	[3]
q	Period (age) preceding initial increase in exposure to vector bites during childhood	0 years	[3]
**Human host infection**			
$\beta = h/g$	Per blackfly biting rate on humans, calculated as the product of the proportion of blackfly bites taken on humans (the human blood index, h) and the reciprocal of the duration of the gonotrophic cycle, g	$h = 0.63$§ $g = 1/104$ years	[2, 4]
$ABR = \beta V/H$	Annual biting rate of blackflies on humans; the key variable for simulating different endemicity levels	Varies; bites/person/year	[1]
$ATP(t) = ABR \times L3(t)$	Annual transmission potential of blackflies to humans	Defined by ABR and $L3(t)$	[2]
$\Pi_{H(i)}[ATP(t-\tau_H), \Omega_T(a_{(i)}-\tau_H)] = \left[\frac{\delta_{H0} + \delta_{H\infty} c_H ATP(t-\tau_H)\Omega_T(a_{(i)}-\tau_H)}{1 + c_H ATP(t-\tau_H)\Omega_T(a_{(i)}-\tau_H)}\right]$	Density-dependent constraint on the proportion of infective L3 larvae successfully establishing as adult worms	defined by δ_{H0}, $\delta_{H\infty}$, c_H, $ATP(t-\tau_H)$ and $\Omega_T(a_{(i)}-\tau_H)$	[2, 3, 5]

Parameter or variable	Definition	Value and units	Reference
δ_{H0}	Proportion of L3 larvae developing to the adult stage within the human host, per bite, when $ATP(t) \to 0$	Dimensionless, 0.186	Re-estimated in [1]
$\delta_{H\infty}$	Proportion of L3 larvae developing to the adult stage within the human host, per bite, when $ATP(t) \to \infty$	Dimensionless, 0.003	Re-estimated in [1]
c_H	Severity of transmission intensity-dependent parasite establishment within humans	0.005	Re-estimated in [1]
τ_H	Time delay between L3 entering the host and establishing as adult worms	0.8 years	[6]
**Parasite demography**			
y_W	Parameter relating mortality rate to age in adult worms	0.1	[1]
d_W	Parameter relating mortality rate to age in adult worms	6.01	[1]
y_M	Parameter relating mortality rate to age in microfilariae	1.09	[1]
d_M	Parameter relating mortality rate to age in microfilariae	1.43	[1]
L_W	Maximum longevity of adult worms	20 years	[7]
L_M	Maximum longevity of microfilariae	2.5 years	[8]
c_{max}	Number of discrete age classes in adult worms and microfilariae	21	[1]
q_M	Duration of each age class for microfilariae	0.125 years	[1]
q_W	Duration of each age class for adult worms	1 year	[1]
ε^*	Per capita rate of production of microfilariae per mg of skin per (fertile) adult female *Onchocerca volvulus* at age zero	1.15 year^{-1}	[9]
ω	Per capita rate of progression from non-fertile to fertile adult female *O. volvulus*	0.59 year^{-1}	[9, 10]
λ_0	Per capita rate of reversion from fertile to non-fertile adult female *O. volvulus*	0.33 year^{-1}	[9, 10]
F	Parameter relating parasite fecundity to age	70	[1]
G	Parameter relating parasite fecundity to age	0.72	[1]

Parameter or variable	Definition	Value and units	Reference
**Larval stages within the vector and adult female blackfly population dynamics.**			
$\Pi_{V(i)}(t) = \dfrac{\delta_{V0}}{\left[1 + c_V M_{(i)}(t)\Omega_T(a_{(i)})\right]}$	Proportion of microfilariae (mf) per mg of skin in human host i developing into infective L3 larvae within the blackfly vector per bite	Defined by δ_{V0}, c_V, $M_{(i)}(t)$ and $\Omega_T(a_{(i)})$	[2, 9]
δ_{V0}	Proportion of mf per mg developing to the infective L3 stage per bite when $M_{(i)}(t) \to 0$	Dimensionless, 0.0207	[9]
c_V	Severity of constraining density-dependent larval development per dermal microfilaria	0.00878	[2]
ν_1	Per capita development rate from L1 to L2 larvae	201.6 year^{-1}	[11]
ν_2	Per capita development rate from L2 to L3 larvae	207.7 year^{-1}	[11]
μ_V	Per capita mortality rate of blackfly vectors	26 year^{-1}	[2, 9]
α_V	Per capita microfilaria-induced mortality of blackfly vectors	0.39 year^{-1}	[2]
τ_V	Delay before L1 larvae can start transitioning to L2 stages	0.011 years (4 days)	[11]
**Ivermectin treatment**			
$\mu'_{M(i)}(\tau_{h(i)}) = (\tau_{h(i)} + u)^{-\kappa}$	Ivermectin-induced per capita rate of excess mortality of microfilariae at time $\tau_{h(i)}$ since treatment	Defined by u and κ	[10]
u	Constant to allow for very large yet finite microfilaricidal effect upon treatment with ivermectin	9.6×10^{-3}	[10]
κ	Shape parameter for excess microfilarial mortality following treatment with ivermectin	1.25	[10]
$\lambda'_{(i)}(\tau_{h(i)}) = \lambda^{max} e^{(-\varphi\tau_{h(i)})}$	Ivermectin-induced per capita rate of reversion from fertile to non-fertile adult female *O. volvulus* at time $\tau_{h(i)}$ since the last treatment	Defined by λ^{max} and φ	[10]
λ^{max}	Maximum rate of ivermectin-induced female worm sterility	32.4 year^{-1}	[10]
φ	Rate of decay of ivermectin-induced female worm sterilisation	19.6 year^{-1}	[10]
λ'_p	Proportion of adult female worms made permanently infertile at each ivermectin treatment round	0.345	[12]
**Skin microfilarial intensity (density) and prevalence**			
$M^*_{(i,k)}(t)$	The observed number of microfilariae in a single skin snip k from human host i	Model output	[1]

Parameter or variable	Definition	Value and units	Reference
w	The average weight of skin for one skin snip (taken with a Holth-type corneoscleral punch [27])	2 mg	[9, 13]
$\bar{M}^*_{(i)}(t)$	The mean number of microfilariae per mg skin from n skin snips of weight w in human host i	Model output	[1]
$\bar{P}^*_{(i)}(t)$	A binary variable indicating positivity for microfilariae in human host i	Model output	[1]
n	The number of skin snips taken per individual human host	2	[1]
$\bar{M}^*(t)$	The mean number of microfilariae per mg of skin per human host	Model output	[1]
$k_{M(i)} = k_{M0} + k_{M1} W_{F(i)}$	The degree of microfilarial aggregation within the skin of human host i	defined by k_{M0} and k_{M1}	[1]
k_{M0}	The degree of microfilarial aggregation in the skin as $W_{F(i)}(t) \to 0$	0.313	[1]
k_{M1}	The change in microfilarial aggregation with increasing $W_{F(i)}(t)$	0.048 per adult female worm	[1]

1.3 Parameters values used to simulate varying pre-control onchocerciasis microfilarial prevalence levels in EPIONCHO-IBM

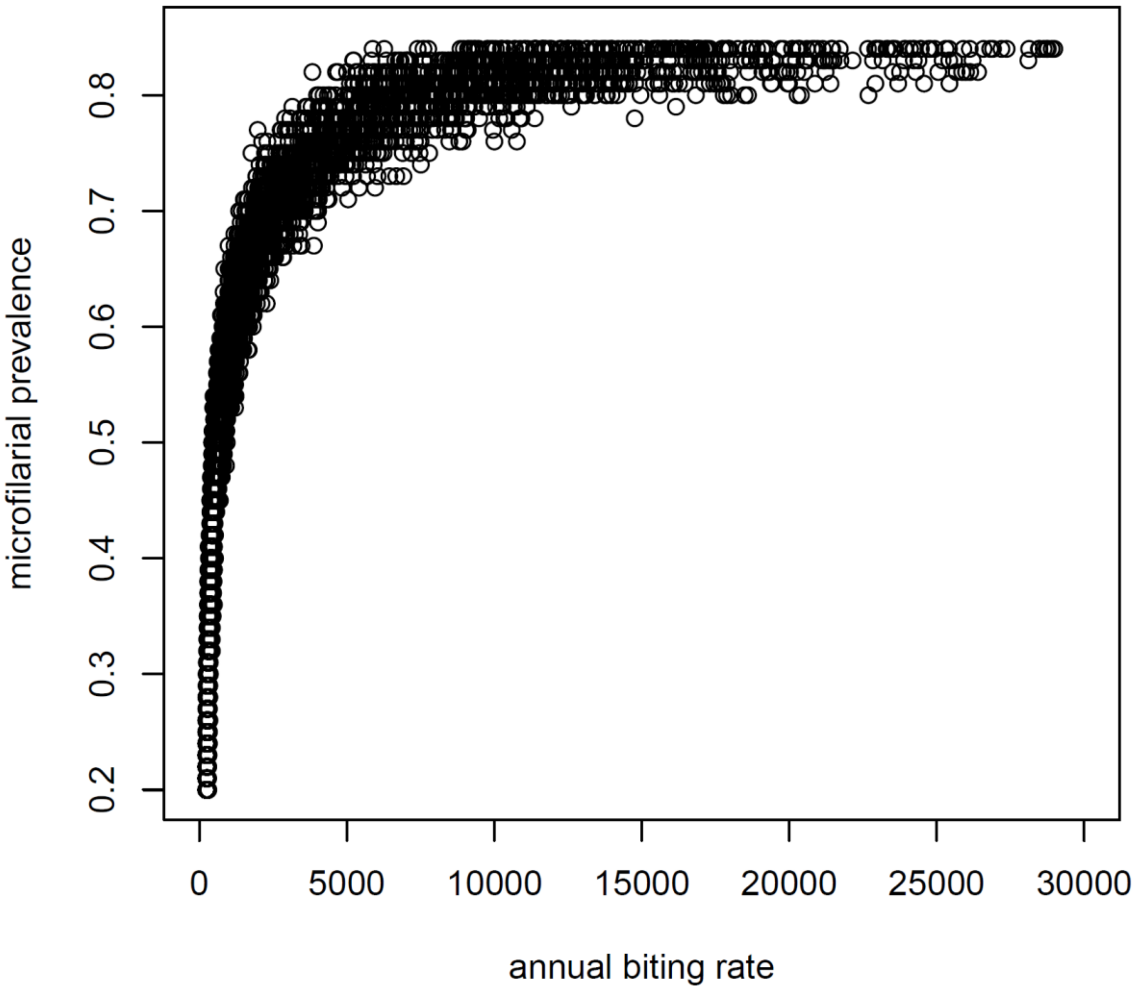

Figure S1. The annual biting rates used to simulate the baseline (pre-control) onchocerciasis microfilarial (mf) prevalence in EPIONCHO-IBM, in each 1% prevalence bin. Each 1%-bin in the lowest (20-25%) and highest (80-85%) bins contain 192 annual biting rates; all other 1%-bins contain 96 (7,200 in total). No other parameters were varied to capture baseline conditions in EPIONCHO-IBM.

Section 2. ONCHOSIM: model and parameter values

2.1 Model version and availability

Model version: WORMSIM2.58Ap27

Simulation programme and programme code: https://gitlab.com/erasmusmc-public-health/wormsim.previous.versions/-/blob/master/wormsim-2.58Ap27.zip.

2.2 Model parameters and their values

Table S2. ONCHOSIM quantification used to simulate onchocerciasis transmission.

Parameter	Value	Source
**Transmission of infection**		
**General transmission parameters**		
Relative biting rate (*rbr*)	Multiplied with the reference *mbr* values, to modify the monthly and annual biting rate: varied between simulations.	
Seasonal variation in contribution to reservoir (*mbr*)	Reference *mbr* values (Jan-Dec): 5340, 4700, 3000, 3840, 3880, 3380, 5260, 6820, 6020, 6580, 7500, 5380	Assumed
Reference value of annual biting rate	(sum of reference *mbr* values for Jan-Dec:: 61,700	
Transmission probability (*v*), i.e. the probability that an infective particle in the reservoir successfully develops into a parasite life stage that is capable of infecting a human host	*v* = 0.07345; see reference for the derivation of this value, given parameters for fly biology and development of infective L3 larvae within the fly.	Coffeng *et al.*[14]
Success ratio (*sr*)	*sr* = 0.0031	Duke[15] & Plaisier[16]
Zoophily (*z, 1 -h*)	*z* = 0.04 ; *h* = 0.96	Habbema *et al.*[17] & expert opinion (OCP entomologists)
**Individual relative exposure and contribution to flies**		
Relative exposure and contribution by age and sex	Zero at birth, linearly increasing between ages 0–20 from 0 to 1.0 for men and from 0 to 0.7 for women, and then constant from the age of 20 years onwards	Plaisier[16]
Variation due to personal factors (fixed through life) given age and sex (α_{Exi})	Gamma distribution with mean 1.0. Shape and rate equal varies between simulations	

Parameter	Value	Source
**Life history and productivity of the parasite in the human host**		
Average worm lifespan (*Tl*)	10 years	Plaisier *et al.*[7]
Variation in worm lifespan	Weibull distribution with shape 3.8.	Assumption; Plaisier *et al.*[7]
Prepatent period (*pp*)	1 year	Plaisier *et al.*[7] which refers to Duke[18] & Prost[6]
Age-dependent microfilaria production capacity, *R(a)*	*R(a)* = 1 for $0 \leq a < 5$<br>*R(a)* = 1-((a-5)/15) for $5 \leq a < 20$<br>*R(a)* = 0 for $a > 20$	Plaisier *et al.*[7] which refers to Albiez[19] & Karam *et al.*[20]
Longevity of microfilariae within host (*Tm*)	9 months	Plaisier[16]
Mating cycle (*rc*)	3 months	Plaisier[16] & Plaisier *et al.*[7] which refers to Schulz-Key[21] & Schulz-Key and Karam[22]
Male potential (*pot*)	100 female worms.	Plaisier [16]
**Density-dependent female worm reproductive capacity**		
Average contribution of an inseminated worm at peak fecundity to the skin mf-density	7.6 mf/worm	Plaisier[16]
Exponential saturation of individual female worm productivity per worm present in host (λ_z)	$\lambda_z = 0$ i.e. no exponential saturation.	Assumption
**Morbidity**		
Disease threshold (*Elc*) for blindness	Weibull distribution with mean 10.000 and shape 2.0	Coffeng *et al.*[23]
Reduction in remaining life expectancy due to blindness (*rl*)	50%	Coffeng *et al.*[23] which refers to partly published data from OCP; Dadzie *et al.*[24];<br>Plaisier *et al.*[25] which refers to Kirkwood *et al.*[26] & Prost and Vaugelade[27]
**Infection dynamics in the vector**		

Parameter	Value	Source
L1-uptake in the vector	Exponential saturating function with parameters *a* = 1.2, *b* = 0.0213, and *c* = 0.0861.	Plaisier *et al.*[28] which refers to Philippon[29] & WHO[30]
**Mass treatment coverage**		
Timing and coverage (C_w)	Varied between scenarios	
Relative compliance (c_r(k, s)) by age and sex		Based on unpublished OCP data

Age-group (k)	0-4	5-9	10-14	15-19	20-29	30-49	50+
**cr(k,males)**	0.00	0.75	0.80	0.80	0.70	0.75	0.80
**cr(k,females)**	0.00	0.75	0.70	0.74	0.65	0.70	0.75

**Drug treatment**		
Proportion of microfilariae cleared from host	100%	Plaisier *et al.*[12]
Duration of temporary reduction in female reproductive capacity (Tr_0), average	11 months	Plaisier *et al.*[12]
Permanent reduction in female worm reproductive capacity (d_0), average	34.9%	Plaisier *et al.*[12]
Proportion of adult worms killed (m_0)	0%	Plaisier *et al.*[12]
Relative effectiveness of treatment in a person (*v*)	Weibull distribution with mean 1 and shape 2	Plaisier *et al.*[12]
Surveys		
Timing	Surveys are done at yearly intervals from 1988-2076. They are always done in month 6, i.e. exactly 12 or 6 months after annual or biannual treatment respectively. The simulation allows for a 200-year warming-up period before the first survey in 1998.	
Dispersal factor for worm contribution to measured density of infective material (*d*)	Exponential distribution with mean 1	Plaisier *et al.*[7]
Variability in measured host load of infective material (here: mf per skin snip)	Poisson distribution with mean $ss(t)$	Plaisier *et al.*[25]

2.3 Parameters combinations used to simulate varying pre-control onchocerciasis microfilarial prevalence levels

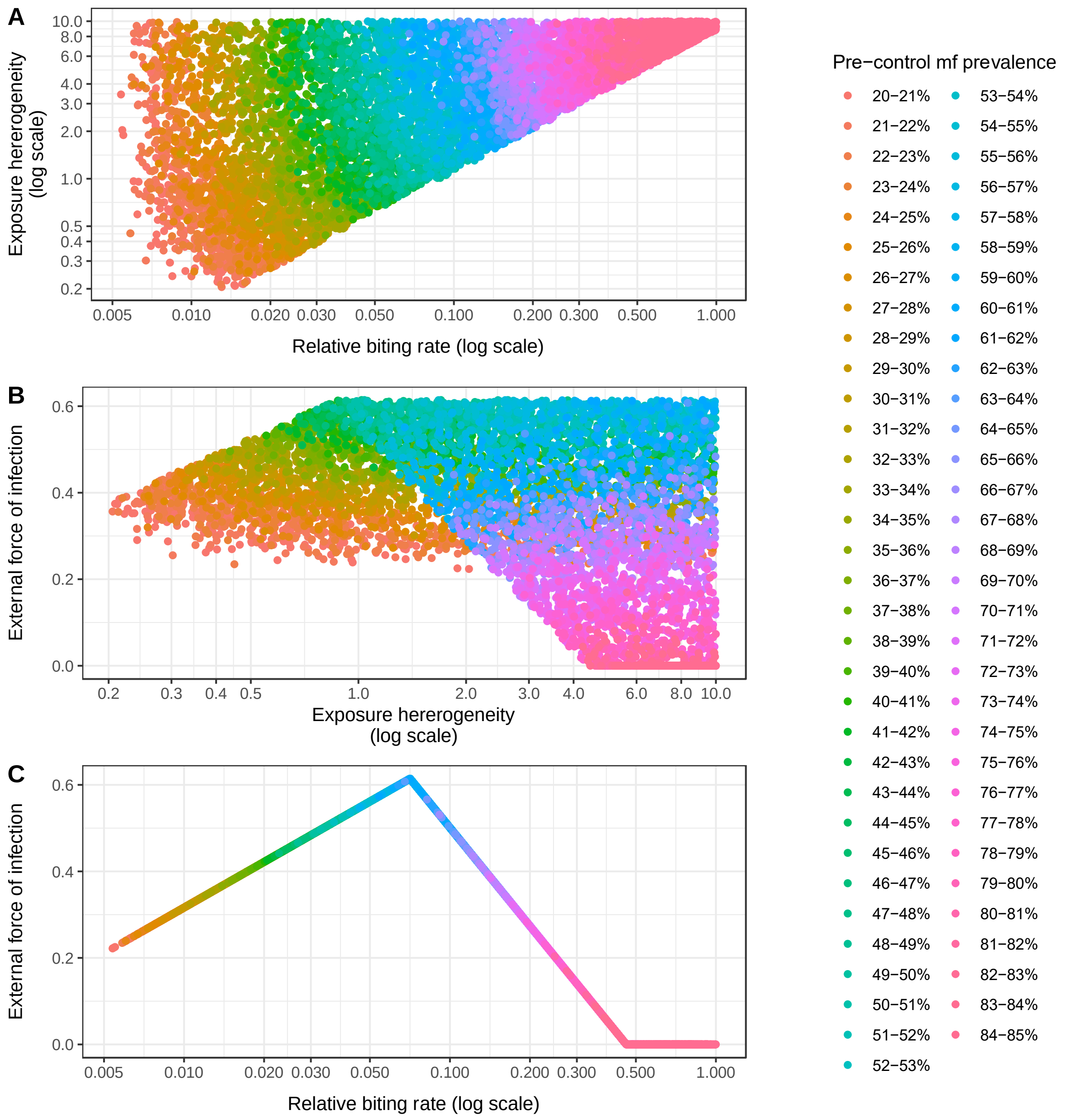

Figure S2. Parameters combinations used to simulate the baseline pre-control onchocerciasis microfilarial (mf) prevalence. Results of sampling the relative biting rate, exposure heterogeneity and external force of infection from a predefined parameter space. Each dot represents the outcome of one simulation run. The colours represent the pre-control onchocerciasis microfilarial prevalence bin to which a parameter combination belongs. Each 1%-bin in the lowest (20-25%) and highest (80-85%) bins contain 200 parameter combinations; all other 1%-bin contains 100 parameter combinations (7,500 in total). The sampled value of the relative biting rate is multiplied with the reference annual biting rate of *61,700* (see Table S2).

Section 3. Project list with assumptions on history of control, population size, and population distribution over endemicity categories

Table S3 list the 123 projects (APOC's MDA implementation units) included in our analysis, modified from the list presented by Kim et al [31]. As the current analysis focussed on the APOC area, former OCP countries were excluded for the current analysis. We further excluded the following areas:

1) Untreated low-endemic areas that were not prioritized for treatment by APOC, but require treatment in view of the current elimination target; most of these remain to be delineated by onchocerciasis elimination mapping [32]. These projects were labelled with "P5" in the project name, in the list of Kim et al.
2) Areas reporting elimination of the vector and/or parasite, i.e. Abu Hamed in Sudan [33], Bioko in Equitorial Guinea [34], and phase 1, 2, 3 and 4 projects in Uganda [35].
3) Areas where MDA was stopped before 2019 as elimination was deemed to have been achieved [36, 37];
4) Meso- and hyperendemic areas identified recently, which were not covered by APOC. These projects were labelled with "P20" in the project name, in the list of Kim et al.
5) Liberia, as it is geographically separate from the rest of APOC.

Table S3. Project list with assumptions on history of control, population size, and population distribution over endemicity categories.

Project identifying information			Assumed history of MDA		Population size in 2015	Total number of pixels	Fraction of pixels in each nodule prevalence category				
Project number	Country	Project name [1]	MDA start year[2]	Frequency of MDA [3]			<10%	10%-20%	20%-40%	40%-60%	>60%
47	Angola	Bengo	2010	1	27,020	5,783	0.314	0.456	0.231	0.000	0.000
49	Angola	Benguela	2012	1	51,685	9,019	0.290	0.350	0.360	0.000	0.000
67	Angola	Cuanza Norte	2011	1	26,413	5,454	0.177	0.499	0.324	0.000	0.000
84	Angola	Huila	2010	1	243,191	27,738	0.157	0.340	0.309	0.168	0.026
103	Angola	Kuando Kubango	2009	1	419,267	105,775	0.389	0.384	0.227	0.000	0.000
111	Angola	Lunda Norte	2009	1	313,245	99,789	0.253	0.315	0.407	0.025	0.000
112	Angola	Lunda sul	2009	1	263,916	90,863	0.308	0.360	0.276	0.056	0.000
121	Angola	Moxico 1	2011	1	275,814	58,856	0.350	0.409	0.238	0.004	0.000
122	Angola	Namibe[4]	2014	1	36,472	5,652	0.179	0.583	0.198	0.040	0.000
48	Angola	NY Benguela	2014	1	116,100	32,822	0.346	0.325	0.280	0.049	0.000
66	Angola	NY Cuanza Norte	2014	1	18,942	5,405	0.193	0.746	0.061	0.000	0.000
83	Angola	NY Huila	2014	1	23,505	6,418	0.164	0.582	0.200	0.054	0.000
110	Angola	NY Lunda Norte	2014	1	68,242	19,379	0.327	0.435	0.238	0.000	0.000
120	Angola	NY Moxico 1	2014	1	352,070	64,230	0.292	0.549	0.159	0.000	0.000
161	Angola	Uige	2014	1	193,601	54,749	0.129	0.437	0.408	0.025	0.000
170	Angola	Zaire	2014	1	14,602	4,163	0.224	0.633	0.144	0.000	0.000
53	Burundi	Bururi	2008	1	379,920	1,112	0.010	0.611	0.379	0.000	0.000
63	Burundi	Cibitoke-Bubanza	2006	1	907,210	2,385	0.117	0.314	0.428	0.141	0.000
138	Burundi	Rutana	2008	1	295,768	1,548	0.218	0.592	0.191	0.000	0.000
38	Cameroon	Adamaoua 1	2008	1	511,231	41,694	0.143	0.358	0.342	0.107	0.050
39	Cameroon	Adamaoua 2	2004	1	464,531	33,595	0.193	0.229	0.372	0.170	0.036
59	Cameroon	Centre 1 [5]	2005	1	472,128	37,361	0.000	0.081	0.295	0.294	0.330
60	Cameroon	Centre 2	2005	1	111,129	14,225	0.152	0.145	0.275	0.236	0.193

Project identifying information			Assumed history of MDA		Population size in 2015	Total number of pixels	Fraction of pixels in each nodule prevalence category				
Project number	Country	Project name [1]	MDA start year[2]	Frequency of MDA [3]			<10%	10%-20%	20%-40%	40%-60%	>60%
61	Cameroon	Centre 3	2004	1	358,824	10,193	0.045	0.289	0.301	0.276	0.089
68	Cameroon	East	2007	1	131,058	18,826	0.139	0.245	0.276	0.167	0.174
77	Cameroon	Far North	2007	1	309,972	3,173	0.345	0.450	0.204	0.000	0.000
105	Cameroon	Littoral 1	2007	1	309,898	3,122	0.000	0.083	0.271	0.271	0.375
106	Cameroon	Littoral 2 [5]	2006	1	165,320	12,745	0.000	0.035	0.129	0.309	0.528
125	Cameroon	Northern	2004	1	695,067	47,895	0.057	0.180	0.385	0.188	0.191
126	Cameroon	Northwest	2005	1	903,003	23,569	0.000	0.113	0.447	0.383	0.057
144	Cameroon	South	2006	1	330,023	27,683	0.201	0.156	0.516	0.127	0.000
145	Cameroon	South West 1	2005	1	432,685	10,332	0.035	0.034	0.048	0.436	0.446
146	Cameroon	South West 2	2004	1	294,465	12,811	0.025	0.034	0.170	0.547	0.224
168	Cameroon	Western	2003	1	1,809,161	14,498	0.000	0.000	0.024	0.233	0.742
55	CAR	CAR region 3 [6]	1996	1	441,047	89,863	0.004	0.012	0.092	0.629	0.263
56	CAR	CAR region 4 [6]	1997	1	459,521	80,462	0.103	0.238	0.341	0.248	0.071
57	CAR	CAR region 5 [6]	1998	1	425,081	58,429	0.402	0.104	0.357	0.121	0.016
58	CAR	CAR region 6 [6]	2003	1	654,065	95,393	0.125	0.062	0.241	0.422	0.151
62	Chad	Chad	2001	1	2,210,586	96,325	0.092	0.309	0.478	0.113	0.009
64	Congo	Congo 1 [5]	2008	1	913,359	28,051	0.132	0.440	0.364	0.052	0.012
43	DRC	Bandundu	2005	1	6,575	44,006	0.607	0.161	0.168	0.061	0.003
44	DRC	Bas-Congo Kinshasa	2008	1	1,556,831	36,862	0.096	0.208	0.393	0.271	0.032
54	DRC	Butembo-Beni	2011	1	967,353	11,998	0.000	0.083	0.552	0.282	0.083
76	DRC	Equateur-Kiri	2009	1	1,285,619	76,480	0.033	0.094	0.574	0.254	0.046
87	DRC	Ituri-Nord	2009	1	1,300,847	8,472	0.003	0.064	0.073	0.194	0.666
88	DRC	Ituri-Sud	2012	1	1,189,111	54,797	0.080	0.060	0.116	0.159	0.585
95	DRC	Kasai	2009	1	11,153,456	217,450	0.203	0.225	0.258	0.157	0.156

Project identifying information			Assumed history of MDA		Population size in 2015	Total number of pixels	Fraction of pixels in each nodule prevalence category				
Project number	Country	Project name [1]	MDA start year[2]	Frequency of MDA [3]			<10%	10%-20%	20%-40%	40%-60%	>60%
96	DRC	Kasongo	2009	1	1,397,198	73,614	0.290	0.249	0.308	0.102	0.050
98	DRC	Katanga-Nord	2009	1	649,132	22,579	0.172	0.230	0.301	0.130	0.166
99	DRC	Katanga-Sud	2009	1	719,004	52,220	0.181	0.250	0.262	0.149	0.158
108	DRC	Lualaba	2008	1	233,221	19,826	0.016	0.229	0.421	0.248	0.086
109	DRC	Lubutu	2009	1	346,439	30,119	0.000	0.000	0.103	0.743	0.155
116	DRC	Masisi-Walikale	2010	1	1,085,812	18,320	0.033	0.046	0.269	0.425	0.226
118	DRC	Mongala	2009	1	1,509,864	44,294	0.065	0.125	0.608	0.150	0.052
97	DRC	NY Katanga-Nord	2014	1	471,020	22,832	0.730	0.117	0.114	0.028	0.012
107	DRC	NY Lualaba	2014	1	1,019,358	49,238	0.519	0.206	0.199	0.076	0.000
115	DRC	NY Masisi-Walikale	2014	1	56,585	2,196	0.190	0.156	0.491	0.163	0.000
139	DRC	NY Rutshuru-Ngoma	2014	1	8,808	294	0.000	0.000	0.282	0.718	0.000
142	DRC	NY Sankuru	2014	1	486,324	23,486	0.202	0.472	0.235	0.047	0.045
159	DRC	NY Ueles	2014	1	165,447	8,001	0.000	0.078	0.158	0.187	0.577
140	DRC	Rutshuru-Ngoma	2009	1	683,884	957	0.000	0.226	0.774	0.000	0.000
143	DRC	Sankuru	2007	1	1,106,393	69,115	0.111	0.230	0.148	0.129	0.382
153	DRC	Tshopo	2010	1	1,652,750	181,335	0.029	0.094	0.246	0.328	0.304
154	DRC	Tshuapa	2010	1	1,472,510	128,064	0.032	0.099	0.253	0.366	0.250
157	DRC	Ubangi-Nord	2011	1	829,146	50,831	0.008	0.127	0.365	0.185	0.314
158	DRC	Ubangi-Sud	2011	1	1,398,580	34,076	0.073	0.330	0.460	0.130	0.007
160	DRC	Ueles	2006	1	1,631,034	161,754	0.020	0.031	0.148	0.216	0.584
42	Ethiopia	Assosa	2014	2	581,826	13,683	0.092	0.525	0.344	0.039	0.000
46	Ethiopia	Bench-Maji	2005	1>2	769,319	12,228	0.000	0.123	0.564	0.307	0.005
72	Ethiopia	East Wellega	2006	1>2	942,125	8,236	0.026	0.336	0.483	0.141	0.014
79	Ethiopia	Gambella	2006	1>2	112,558	15,702	0.019	0.317	0.416	0.247	0.001

Project identifying information			Assumed history of MDA		Population size in 2015	Total number of pixels	Fraction of pixels in each nodule prevalence category				
Project number	Country	Project name [1]	MDA start year[2]	Frequency of MDA [3]			<10%	10%-20%	20%-40%	40%-60%	>60%
82	Ethiopia	Horo Guduru	2014	2	55,244	1,295	0.000	0.225	0.707	0.068	0.000
85	Ethiopia	Illubabor	2004	1>2	796,862	17,708	0.001	0.095	0.652	0.211	0.041
90	Ethiopia	Jimma	2004	1>2	940,627	16,706	0.125	0.425	0.415	0.034	0.000
92	Ethiopia	Kaffa-Sheka	2003	1>2	1,335,929	13,720	0.122	0.091	0.237	0.512	0.037
93	Ethiopia	Kamashi	2014	2	496,800	11,392	0.020	0.167	0.546	0.189	0.078
117	Ethiopia	Metekel	2007	1>2	170,027	14,429	0.163	0.276	0.487	0.074	0.000
124	Ethiopia	North Gondar	2004	1>2	330,145	23,227	0.314	0.294	0.311	0.075	0.007
71	Ethiopia	NY East Wellega	2014	2	301,569	7,082	0.004	0.054	0.710	0.205	0.028
166	Ethiopia	NY West Wellega	2014	2	306,292	7,127	0.080	0.349	0.571	0.000	0.000
165	Ethiopia	West Shewa	2014	2	60,899	1,433	0.214	0.624	0.163	0.000	0.000
167	Ethiopia	West Wellega	2006	1>2	1,081,831	12,812	0.039	0.157	0.398	0.308	0.097
114	Malawi	Malawi Extension	2004	1	1,340,321	4,861	0.193	0.553	0.254	0.000	0.000
152	Malawi	Thyolo Mwanza	2004	1	1,022,249	4,664	0.024	0.268	0.708	0.000	0.000
40	Nigeria	Adamawa	2001	1	1,894,700	36,280	0.625	0.184	0.179	0.012	0.000
41	Nigeria	Akwa Ibom	2006	1	32,439	919	0.157	0.637	0.207	0.000	0.000
45	Nigeria	Bauchi	2009	1	1,941,246	37,004	0.498	0.389	0.110	0.003	0.000
50	Nigeria	Benue	2007	1	3,828,500	35,655	0.058	0.151	0.537	0.210	0.045
52	Nigeria	Borno	2006	1	1,526,062	33,551	0.183	0.529	0.288	0.000	0.000
65	Nigeria	Cross River [5]	2003	1	1,383,685	19,731	0.166	0.360	0.324	0.105	0.045
73	Nigeria	Edo Delta [5]	2006	1	1,778,941	24,884	0.131	0.336	0.499	0.034	0.000
74	Nigeria	Ekiti [5]	2007	1	2,401,544	6,178	0.000	0.837	0.163	0.000	0.000
75	Nigeria	Enugu Anambra Ebony	1999	1	2,643,388	17,804	0.004	0.044	0.369	0.471	0.113
78	Nigeria	FCT	2004	1	561,180	8,675	0.346	0.584	0.071	0.000	0.000
80	Nigeria	Gombe	2006	1	2,109,353	21,476	0.313	0.493	0.169	0.026	0.000

Project identifying information			Assumed history of MDA		Population size in 2015	Total number of pixels	Fraction of pixels in each nodule prevalence category				
Project number	Country	Project name [1]	MDA start year[2]	Frequency of MDA [3]			<10%	10%-20%	20%-40%	40%-60%	>60%
86	Nigeria	Imo Abia	1999	1	1,457,213	7,066	0.027	0.467	0.375	0.130	0.000
89	Nigeria	Jigawa	2004	1	395,032	11,708	0.742	0.249	0.010	0.000	0.000
94	Nigeria	Kano	2000	1	1,095,220	12,846	0.297	0.581	0.122	0.001	0.000
100	Nigeria	Kebbi	2006	1	230,134	10,019	0.605	0.340	0.055	0.000	0.000
102	Nigeria	Kogi [5]	2005	1	1,977,483	31,631	0.106	0.464	0.401	0.030	0.000
104	Nigeria	Kwara [5]	2003	1	1,671,362	36,671	0.016	0.458	0.508	0.018	0.000
123	Nigeria	Niger	2004	1	2,938,072	73,279	0.103	0.687	0.201	0.008	0.001
128	Nigeria	Ogun	2003	1	380,642	9,597	0.000	0.432	0.559	0.009	0.000
129	Nigeria	Ondo [5]	2007	1	1,514,082	10,899	0.004	0.373	0.623	0.000	0.000
130	Nigeria	Osun	2009	1	1,786,284	10,569	0.003	0.224	0.773	0.000	0.000
131	Nigeria	Oyo	2011	1	1,218,220	27,320	0.000	0.436	0.564	0.000	0.000
151	Nigeria	Taraba [5]	2003	1	1,842,949	50,020	0.172	0.343	0.312	0.134	0.039
169	Nigeria	Yobe	2002	1	681,616	35,027	0.268	0.640	0.092	0.000	0.000
171	Nigeria	Zamfara	1999	1	316,334	2,424	0.446	0.530	0.024	0.000	0.000
69	South Sudan	East Bahr El Ghazal	2011	1	603,984	41,331	0.003	0.145	0.722	0.116	0.015
70	South Sudan	East Equatoria	2009	1	1,073,560	60,463	0.084	0.208	0.543	0.158	0.007
162	South Sudan	Upper Nile	2010	1	562,551	54,619	0.346	0.408	0.211	0.034	0.001
163	South Sudan	West Bahr El Ghazal	2011	1	3,255,512	163,632	0.161	0.283	0.439	0.060	0.056
164	South Sudan	West Equatoria	2009	1	767,891	84,524	0.108	0.193	0.516	0.170	0.013
149	Sudan	Sudan	2008	1	221,430	9,437	0.992	0.008	0.000	0.000	0.000
101	Tanzania	Kilosa [5]	2007	1	569,750	8,155	0.465	0.316	0.189	0.030	0.000
113	Tanzania	Mahenge	2003	1	569,616	22,674	0.003	0.123	0.523	0.200	0.151
119	Tanzania	Morogoro [5]	2007	1	407,720	7,604	0.161	0.320	0.464	0.054	0.000
141	Tanzania	Ruvuma	2002	1	450,107	55,156	0.001	0.047	0.229	0.297	0.427

Project identifying information			Assumed history of MDA		Population size in 2015	Total number of pixels	Fraction of pixels in each nodule prevalence category				
Project number	Country	Project name [1]	MDA start year[2]	Frequency of MDA [3]			<10%	10%-20%	20%-40%	40%-60%	>60%
150	Tanzania	Tanga	2004	1	353,122	9,678	0.526	0.169	0.304	0.000	0.000
155	Tanzania	Tukuyu	2001	1	129,227	4,124	0.102	0.196	0.355	0.263	0.084
156	Tanzania	Tunduru	2005	1	140,128	22,200	0.000	0.066	0.446	0.381	0.107
136	Uganda	Phase 5 [7]	2012	2	570,199	14,878	0.523	0.147	0.177	0.128	0.025

Abbreviations: CAR = Central African Republic, DRC = Democratic Republic of Congo, FCT = Federal Capital Territory, NY = not yet

Footnotes:

[1] Project name: NY = not yet. This prefix is used if treatment had not yet started by 2013. We assume that treatment was started in 2014.

[2] First year in which MDA coverage in the area was >=60%

[3] Frequency of MDA: 1 = annual, 2= biannual, 1>2 = switch from annual to biannual (assumption: biannual from 2015 onward).

[4] Namibe, Angola was not included in the project-list from Kim et al.

[5] Start year modified from Kim et al. based on recent insights.

[6] For the purpose of this project, we distinguished 4 subprojects in the combined CAR project with different start years.

[7] Madi Mid North focus in Uganda

Section 4. Methods for estimating the population requiring treatment by project

Step 1: Converting nodule prevalence to microfilarial prevalence
We sampled an microfilarial prevalence value for each pixel per project as follows:
a) The nodule prevalence of a pixel was sampled from the data using the nodule prevalence estimate and standard error [38];
b) The sampled nodule prevalence was converted to a corresponding microfilarial prevalence from a posterior distribution of a previously published model [39]
Figure S3 provides an example of this process.

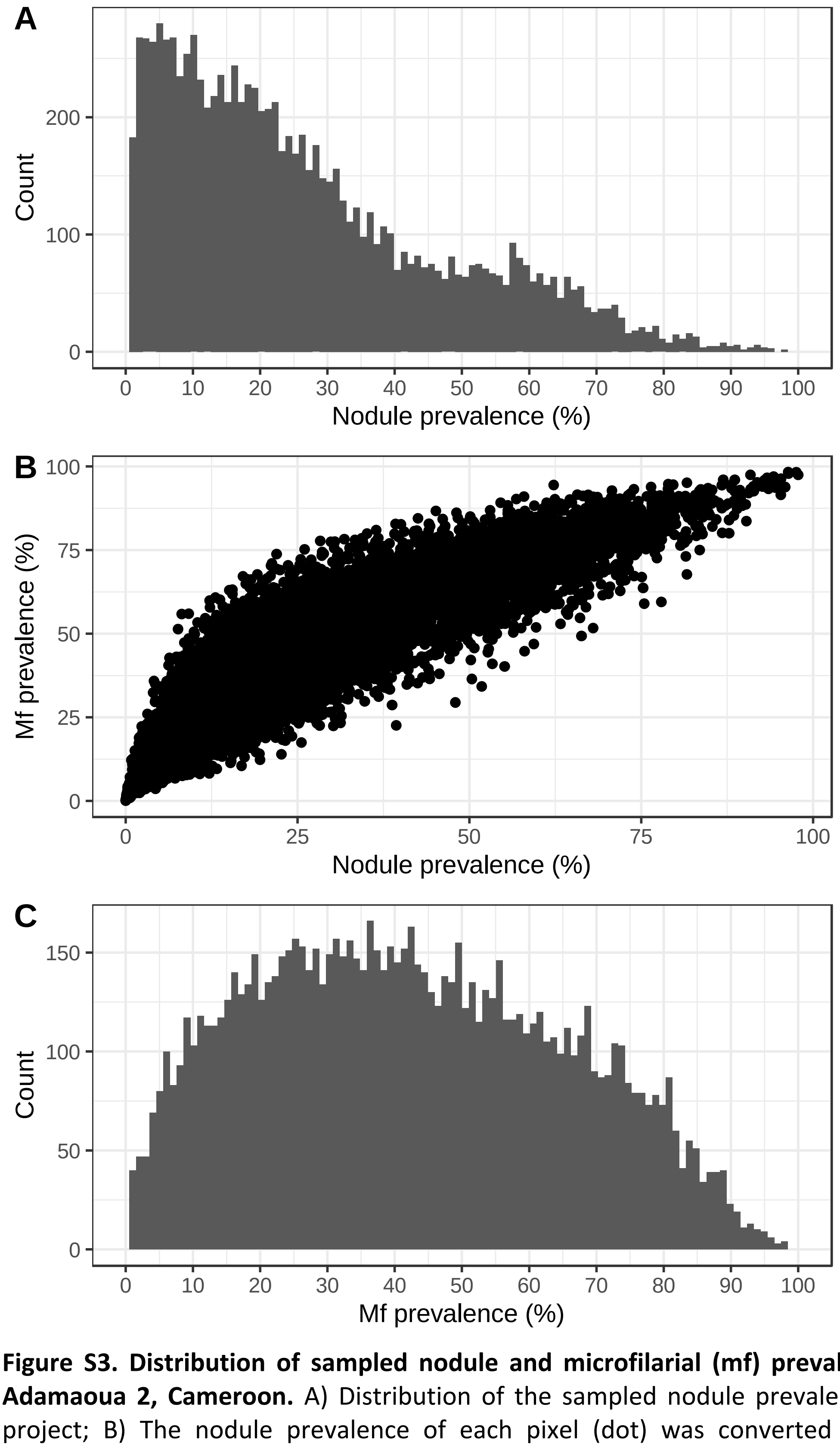

Figure S3. Distribution of sampled nodule and microfilarial (mf) prevalence per pixel in Adamaoua 2, Cameroon. A) Distribution of the sampled nodule prevalence per pixel in a project; B) The nodule prevalence of each pixel (dot) was converted to a microfilarial prevalence; C) Corresponding distribution of microfilarial prevalence per pixel in a project.

Step 2: Assigning simulations to the sampled microfilarial prevalence

The simulations conducted by EPIONCHO-IBM and ONCHOSIM covered pre-control prevalence levels from 20% to 85% per project. All simulation runs for a given project and scenario were binned based on the simulated baseline endemicity (bin-width = 1% microfilarial prevalence). The lowest (20-25%) and highest (80-85%) 1%- microfilarial prevalence bin included 192 and 200 runs per 1%-bin for EPIONCHO-IBM and ONCHOSIM, respectively, while the remaining prevalence range (25-80%) included 96 and 100 runs per 1%-bin, respectively. In total, there were 7,680 (EPIONCHO-IBM) or 7,500 (ONCHOSIM) simulation runs.

For each microfilarial prevalence per pixel of a project, we matched the sampled microfilarial prevalence to a corresponding 1%-bin, and then randomly selected one simulation run from all the runs in the matched 1%-bin from EPIONCHO-IBM and ONCHOSIM. If the microfilarial prevalence was below 20% or above 85%, we selected a simulation from the 20%- or 85%-bin, respectively. For each selected run, we assessed whether elimination would be reached. An example of the results of one project is shown in Figure S4. Step 1 and 2 were repeated 100 times.

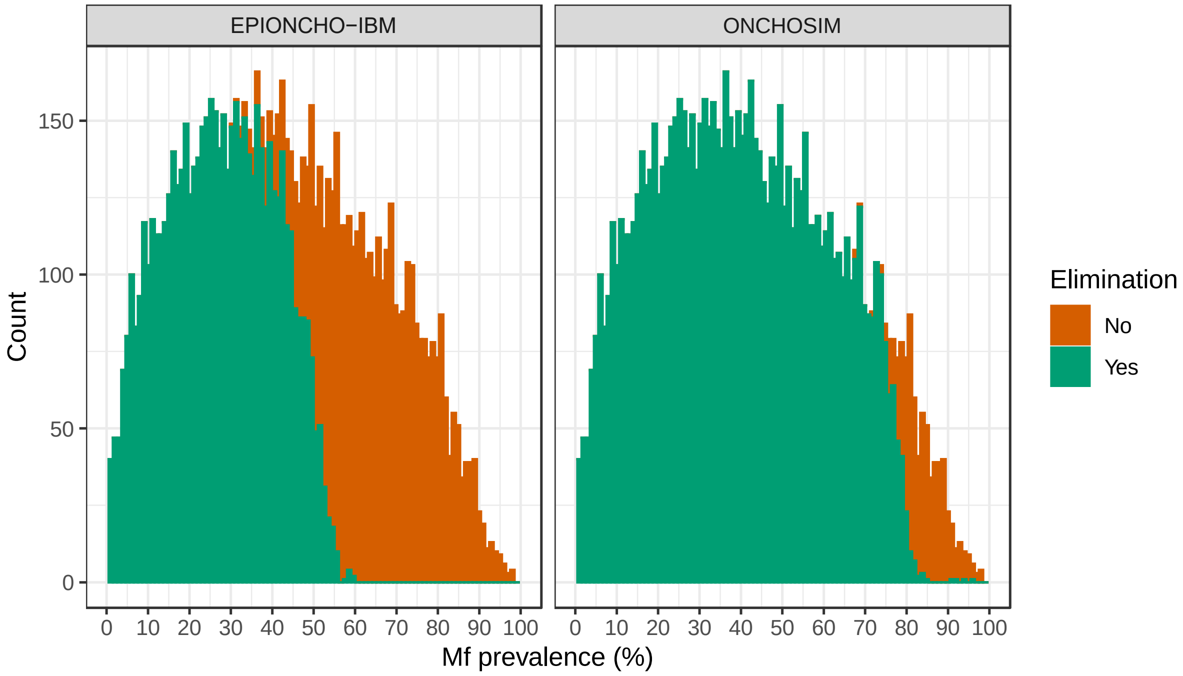

Figure S4. Simulations at pixel level in Adamaoua 2, Cameroon in 2030. This is the result of one iteration. Annual ivermectin MDA from 2004 to 2030. Proportion of pixels that reached elimination in 2030 was 60% for EPIONCHO-IBM and 93% for ONCHOSIM.

Step 3: Calculating population requiring treatment

For each iteration of the previous step, we assessed which proportion of sampled simulation runs for ONCHOSIM and EPIONCHO-IBM resulted in elimination. For example, in figure S2 60%

and 93% of the pixels reached elimination in a project using EPIONCHO-IBM and ONCHOSIM, respectively. The population requiring treatment based on a stop-MDA decision made at project- or community level was calculated as follows:

Decision at project-level: We calculated the proportion of the 100 iterations that reached elimination in at least 90% of the pixels in a project. If this proportion was below 1, the population requiring treatment would equal the annual project-specific average population size of the project. If this proportion was 1, we assumed that elimination was reached in that project and therefore treatment could be stopped.

Decision at community-level: We calculated the mean proportion that resulted in elimination from the 100 iterations per project. The population still requiring treatment was then calculated by multiplying the number of pixels that has not yet achieved elimination by the annual project-specific average population size per pixel.

Figure S5 shows the population requiring treatment in one project. The total number of people requiring treatment was based on the sum of the population requiring treatment of all projects.

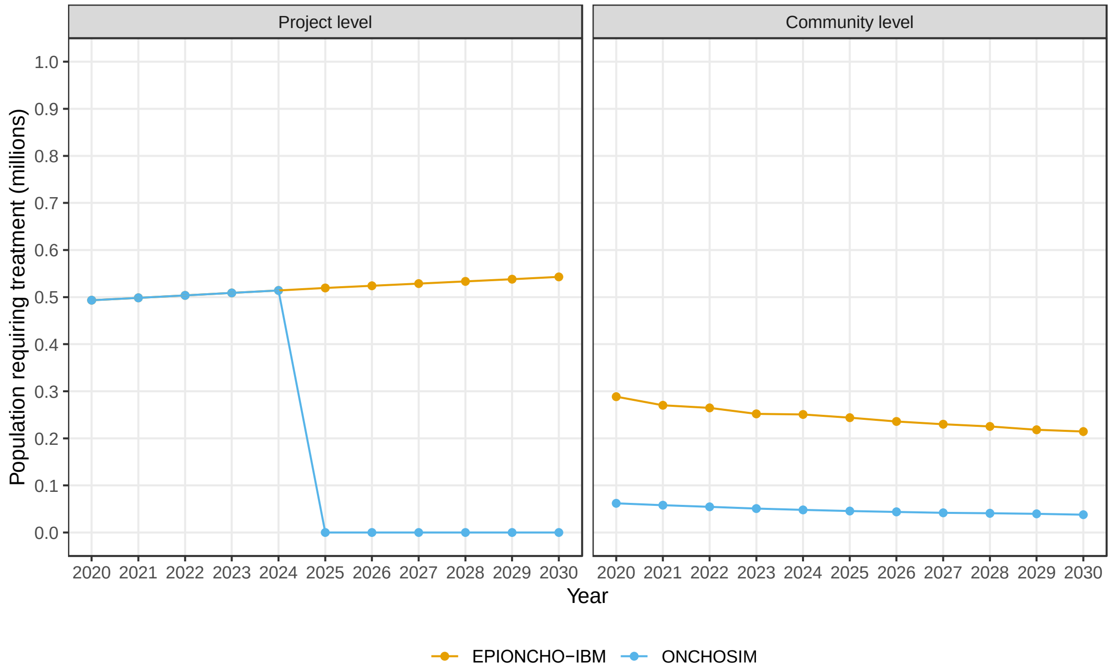

Figure S5. Population requiring treatment by geographical decision scale in Adamaoua 2, Cameroon. Annual ivermectin MDA started in 2004.

Section 5. The Policy-Relevant Items for Reporting Models in Epidemiology of Neglected Tropical Diseases

Table S4. The Policy-Relevant Items for Reporting Models in Epidemiology of Neglected Tropical Diseases (PRIME-NTD) [a]

Principle [b]	What has been done to satisfy the principle?	Where in the manuscript is this described?
1. Stakeholder engagement	Policy makers at global level are key stakeholders, and a representative of that group is included in the author list of this paper (PTC). Our work is relevant for a much wider group of stakeholders, including the many public and private parties involved in, or supporting, the implementation of elimination programmes. During the execution of this work, we have had regular online meetings with a small group of peopled steering research & development to accelerate onchocerciasis elimination.	Author list, author contribution, acknowledgements section
2. Complete model documentation	The paper contains a brief description of the model with references to original model description papers. The model source code is publicly available. We provide full information on the quantification of all model parameters pertaining to transmission, life history and productivity of the parasite, morbidity, vector, drug efficacy, treatment histories (timing, frequency and coverage of mass treatment), and surveys.	Methods section, Supplementary Material section 1 and 2
3. Complete description of data used	Key data inputs for our analyses included 1) data on treatment history by project and 2) high-resolution geospatial data on baseline endemicity within projects. These data have been published previously, and we provide references to these earlier publications. We updated the previously published treatment histories by project, based on insights from recent evaluations and recent treatment data. Our updated assumptions are reported in the Supplementary Material to this paper. We further provide summary information on the geospatial variation in baseline endemicity by projects.	Methods section, Supplementary Material section 3 and 4
4. Communicating uncertainty	We account for uncertainty in the parameters which determine baseline microfilarial prevalence (e.g. annual biting rate, heterogeneity in exposure to vector bites) and for stochastic variation. Structural uncertainty is accounted for by making predictions with two models that differ in their structural assumptions regarding regulation of infection processes in humans and age- and sexspecific exposure patterns. Uncertainty in the assumptions regarding the history of interventions is acknowledged in the manuscript.	Results section 1-3, Discussion, Supplementary Material sections 4 and 5.
5. Testable model outcomes	Model outcomes include the expected year in which elimination is achieved at project-level. The latter can be compared in the future to the actual moment of stopping in ‘projects’.	Supplement 4

[a] Communication of adherence to the five principles of the NTD Modelling Consortium for policy-relevant work, described in: Behrend et al. 2020. Modelling for policy: The five principles of the Neglected Tropical Diseases Modelling Consortium. *PLoS Negl Trop Dis* 2020; **14**(4): e0008033.

[b] Full formulation of the principles:

1. Don't do it alone. Engage stakeholders throughout, from the formulation of questions to the discussions on the implications of the findings.

2. Reproducibility is key! Prepare and make available (preferably open-source) a complete technical documentation of all model code, mathematical formulas, assumptions and their justification, allowing others to reproduce the model.
3. Model calibration, goodness-of-fit and validation are fundamental processes of scientific modelling. All data used should be described in sufficient detail to allow the reader to assess the type and quality of these analyses. When using data by reference, use Principle 2.
4. Communicating uncertainty is a hallmark of good modelling practice. Perform a sensitivity analysis of all key parameters, and for each paper reporting model predictions include an uncertainty assessment of those model outputs within the paper.
5. Model outcomes should be articulated in the form of testable hypotheses. This allows comparison with other models and future events as part of the ongoing cycle of model improvement.

Section 6. Results disaggregated by project

Table S5. Project list with model-predicted estimates of proportion of pixels (or communities) achieving elimination by 2020, 2025, and 2030, and the estimated year in which elimination is achieved at project-level

			EPIONCHO-IBM				ONCHOSIM			
			Communtity-level results: % communities having achieved elimination by year			Year in which elimination is achieved at project level	Communtity-level results: % communities having achieved elimination by year			Year in which elimination is achieved at project level
ID	Country	Project_name	2020	2025	2030	MDA	2020	2025	2030	MDA
47	Angola	Bengo	0.04	0.09	0.15	>2030	0.48	0.62	0.72	>2030
49	Angola	Benguela	0.18	0.59	0.75	>2030	0.95	0.98	0.99	<2020
67	Angola	Cuanza Norte	0.34	0.77	0.90	>2030	0.99	1.00	1.00	<2020
84	Angola	Huila	0.28	0.51	0.61	>2030	0.85	0.90	0.93	2025
103	Angola	Kuando Kubango	0.47	0.66	0.75	>2030	0.93	0.96	0.98	<2020
111	Angola	Lunda Norte	0.49	0.69	0.80	>2030	0.97	0.99	0.99	<2020
112	Angola	Lunda sul	0.39	0.57	0.68	>2030	0.91	0.95	0.97	<2020
121	Angola	Moxico 1	0.27	0.61	0.73	>2030	0.94	0.97	0.99	<2020
122	Angola	Namibe	0.02	0.38	0.57	>2030	0.88	0.94	0.97	2022
48	Angola	NY Benguela	0.02	0.51	0.70	>2030	0.94	0.97	0.98	<2020
66	Angola	NY Cuanza Norte	0.03	0.57	0.80	>2030	0.98	0.99	1.00	<2020
83	Angola	NY Huila	0.02	0.44	0.63	>2030	0.89	0.93	0.96	2021
110	Angola	NY Lunda Norte	0.02	0.38	0.56	>2030	0.86	0.91	0.95	2024
120	Angola	NY Moxico 1	0.02	0.39	0.56	>2030	0.85	0.90	0.94	2025
161	Angola	Uige	0.01	0.27	0.45	>2030	0.86	0.92	0.97	2023
170	Angola	Zaire	0.02	0.44	0.66	>2030	0.95	0.98	0.99	<2020
53	Burundi	Bururi	0.68	0.87	0.94	2028	1.00	1.00	1.00	<2020
63	Burundi	Cibitoke-Bubanza	0.17	0.28	0.36	>2030	0.78	0.88	0.93	2027
138	Burundi	Rutana	0.56	0.78	0.88	>2030	1.00	1.00	1.00	<2020

			EPIONCHO-IBM				ONCHOSIM			
			Communtity-level results: % communities having achieved elimination by year			Year in which elimination is achieved at project level	Communtity-level results: % communities having achieved elimination by year			Year in which elimination is achieved at project level
**ID**	**Country**	**Project_name**	**2020**	**2025**	**2030**	**MDA**	**2020**	**2025**	**2030**	**MDA**
38	Cameroon	Adamaoua 1	0.12	0.23	0.32	>2030	0.70	0.79	0.84	>2030
39	Cameroon	Adamaoua 2	0.42	0.53	0.61	>2030	0.87	0.91	0.93	2024
59	Cameroon	Centre 1	0.11	0.17	0.23	>2030	0.52	0.61	0.65	>2030
60	Cameroon	Centre 2	0.25	0.33	0.40	>2030	0.75	0.82	0.85	>2030
61	Cameroon	Centre 3	0.19	0.28	0.34	>2030	0.65	0.73	0.77	>2030
68	Cameroon	East	0.37	0.50	0.57	>2030	0.80	0.85	0.88	>2030
77	Cameroon	Far North	0.52	0.72	0.83	>2030	0.99	1.00	1.00	<2020
105	Cameroon	Littoral 1	0.03	0.06	0.10	>2030	0.35	0.44	0.50	>2030
106	Cameroon	Littoral 2	0.02	0.04	0.06	>2030	0.28	0.37	0.43	>2030
125	Cameroon	Northern	0.39	0.50	0.57	>2030	0.82	0.86	0.88	>2030
126	Cameroon	Northwest	0.05	0.10	0.15	>2030	0.54	0.65	0.71	>2030
144	Cameroon	South	0.37	0.49	0.58	>2030	0.90	0.94	0.96	2020
145	Cameroon	South West 1	0.06	0.08	0.09	>2030	0.35	0.50	0.59	>2030
146	Cameroon	South West 2	0.05	0.08	0.11	>2030	0.51	0.64	0.72	>2030
168	Cameroon	Western	0.03	0.06	0.09	>2030	0.55	0.66	0.73	>2030
55	CAR	CAR region 3	0.09	0.12	0.15	>2030	0.80	0.85	0.88	>2030
56	CAR	CAR region 4	0.48	0.53	0.56	>2030	0.85	0.87	0.89	>2030
57	CAR	CAR region 5	0.68	0.72	0.74	>2030	0.92	0.94	0.95	<2020
58	CAR	CAR region 6	0.24	0.29	0.33	>2030	0.70	0.78	0.82	>2030
62	Chad	Chad	0.47	0.58	0.66	>2030	0.97	0.98	0.99	<2020
64	Congo	Congo 1	0.25	0.45	0.60	>2030	0.93	0.97	0.98	<2020
43	DRC	Bandundu	0.82	0.88	0.92	2028	0.99	1.00	1.00	<2020
44	DRC	Bas-Congo Kinshasa	0.26	0.40	0.52	>2030	0.89	0.96	0.98	<2020

			EPIONCHO-IBM				ONCHOSIM			
			Communtity-level results: % communities having achieved elimination by year			Year in which elimination is achieved at project level	Communtity-level results: % communities having achieved elimination by year			Year in which elimination is achieved at project level
**ID**	**Country**	**Project_name**	**2020**	**2025**	**2030**	**MDA**	**2020**	**2025**	**2030**	**MDA**
54	DRC	Butembo-Beni	0.04	0.15	0.27	>2030	0.70	0.82	0.90	>2030
76	DRC	Equateur-Kiri	0.07	0.16	0.29	>2030	0.78	0.88	0.93	2026
87	DRC	Ituri-Nord	0.03	0.05	0.08	>2030	0.21	0.30	0.37	>2030
88	DRC	Ituri-Sud	0.06	0.19	0.26	>2030	0.47	0.54	0.62	>2030
95	DRC	Kasai	0.22	0.33	0.41	>2030	0.66	0.74	0.79	>2030
96	DRC	Kasongo	0.48	0.67	0.76	>2030	0.94	0.96	0.98	<2020
98	DRC	Katanga-Nord	0.33	0.48	0.58	>2030	0.77	0.82	0.86	>2030
99	DRC	Katanga-Sud	0.10	0.16	0.21	>2030	0.42	0.49	0.55	>2030
108	DRC	Lualaba	0.17	0.30	0.41	>2030	0.77	0.86	0.90	>2030
109	DRC	Lubutu	0.03	0.07	0.15	>2030	0.71	0.86	0.93	2027
116	DRC	Masisi-Walikale	0.06	0.12	0.18	>2030	0.49	0.62	0.72	>2030
118	DRC	Mongala	0.10	0.20	0.31	>2030	0.72	0.83	0.88	>2030
97	DRC	NY Katanga-Nord	0.03	0.53	0.71	>2030	0.90	0.93	0.96	2020
107	DRC	NY Lualaba	0.02	0.40	0.55	>2030	0.80	0.87	0.94	2027
115	DRC	NY Masisi-Walikale	0.01	0.23	0.34	>2030	0.76	0.87	0.95	2027
139	DRC	NY Rutshuru-Ngoma	0.00	0.00	0.01	>2030	0.48	0.70	0.89	>2030
142	DRC	NY Sankuru	0.00	0.06	0.13	>2030	0.55	0.69	0.81	>2030
159	DRC	NY Ueles	0.00	0.02	0.05	>2030	0.25	0.33	0.42	>2030
140	DRC	Rutshuru-Ngoma	0.07	0.16	0.29	>2030	0.87	0.97	0.99	2021
143	DRC	Sankuru	0.24	0.34	0.40	>2030	0.59	0.64	0.68	>2030
153	DRC	Tshopo	0.12	0.22	0.28	>2030	0.57	0.67	0.74	>2030
154	DRC	Tshuapa	0.08	0.17	0.24	>2030	0.57	0.69	0.78	>2030
157	DRC	Ubangi-Nord	0.04	0.15	0.24	>2030	0.55	0.64	0.71	>2030

			EPIONCHO-IBM				ONCHOSIM			
			Communtity-level results: % communities having achieved elimination by year			Year in which elimination is achieved at project level	Communtity-level results: % communities having achieved elimination by year			Year in which elimination is achieved at project level
**ID**	**Country**	**Project_name**	**2020**	**2025**	**2030**	**MDA**	**2020**	**2025**	**2030**	**MDA**
158	DRC	Ubangi-Sud	0.16	0.45	0.60	>2030	0.91	0.96	0.98	2020
160	DRC	Ueles	0.06	0.09	0.11	>2030	0.29	0.35	0.39	>2030
42	Ethiopia	Assosa	0.22	0.52	0.74	>2030	0.94	0.98	1.00	<2020
46	Ethiopia	Bench-Maji	0.44	0.60	0.71	>2030	0.97	0.99	0.99	<2020
72	Ethiopia	East Wellega	0.34	0.53	0.66	>2030	0.95	0.98	0.99	<2020
79	Ethiopia	Gambella	0.31	0.42	0.50	>2030	0.83	0.91	0.95	2024
82	Ethiopia	Horo Guduru	0.02	0.11	0.28	>2030	0.69	0.89	0.97	2026
85	Ethiopia	Illubabor	0.31	0.47	0.58	>2030	0.91	0.95	0.97	<2020
90	Ethiopia	Jimma	0.59	0.75	0.84	>2030	0.99	1.00	1.00	<2020
92	Ethiopia	Kaffa-Sheka	0.29	0.38	0.46	>2030	0.90	0.95	0.97	2020
93	Ethiopia	Kamashi	0.04	0.13	0.29	>2030	0.61	0.79	0.89	>2030
117	Ethiopia	Metekel	0.56	0.73	0.82	>2030	0.98	0.99	1.00	<2020
124	Ethiopia	North Gondar	0.43	0.56	0.65	>2030	0.93	0.97	0.98	<2020
71	Ethiopia	NY East Wellega	0.25	0.53	0.71	>2030	0.90	0.97	0.99	2020
166	Ethiopia	NY West Wellega	0.10	0.32	0.58	>2030	0.92	0.99	1.00	<2020
165	Ethiopia	West Shewa	0.52	0.83	0.94	2027	0.99	1.00	1.00	<2020
167	Ethiopia	West Wellega	0.30	0.44	0.53	>2030	0.83	0.90	0.94	2025
114	Malawi	Malawi Extension	0.56	0.75	0.85	>2030	1.00	1.00	1.00	<2020
152	Malawi	Thyolo Mwanza	0.38	0.60	0.73	>2030	0.99	1.00	1.00	<2020
40	Nigeria	Adamawa	0.87	0.91	0.93	2024	0.99	0.99	0.99	<2020
41	Nigeria	Akwa Ibom	0.78	0.91	0.96	2026	1.00	1.00	1.00	<2020
45	Nigeria	Bauchi	0.45	0.65	0.77	>2030	0.97	0.99	0.99	2020
50	Nigeria	Benue	0.15	0.27	0.39	>2030	0.86	0.93	0.96	2022

			EPIONCHO-IBM				ONCHOSIM			
			Communtity-level results: % communities having achieved elimination by year			Year in which elimination is achieved at project level	Communtity-level results: % communities having achieved elimination by year			Year in which elimination is achieved at project level
**ID**	**Country**	**Project_name**	**2020**	**2025**	**2030**	**MDA**	**2020**	**2025**	**2030**	**MDA**
52	Nigeria	Borno	0.41	0.60	0.73	>2030	0.99	1.00	1.00	<2020
65	Nigeria	Cross River	0.20	0.30	0.38	>2030	0.83	0.88	0.90	>2030
73	Nigeria	Edo Delta	0.49	0.67	0.77	>2030	0.99	1.00	1.00	<2020
74	Nigeria	Ekiti	0.60	0.82	0.93	2028	1.00	1.00	1.00	<2020
75	Nigeria	Enugu Anambra Ebony	0.07	0.11	0.14	>2030	0.65	0.72	0.77	>2030
78	Nigeria	FCT	0.84	0.93	0.97	2022	1.00	1.00	1.00	<2020
80	Nigeria	Gombe	0.76	0.88	0.93	>2030	0.99	1.00	1.00	<2020
86	Nigeria	Imo Abia	0.67	0.76	0.81	>2030	0.99	1.00	1.00	<2020
89	Nigeria	Jigawa	0.90	0.95	0.98	2020	1.00	1.00	1.00	<2020
94	Nigeria	Kano	0.84	0.92	0.95	2024	1.00	1.00	1.00	<2020
100	Nigeria	Kebbi	0.84	0.94	0.97	2022	1.00	1.00	1.00	<2020
102	Nigeria	Kogi	0.80	0.89	0.94	2026	1.00	1.00	1.00	<2020
104	Nigeria	Kwara	0.35	0.54	0.68	>2030	0.99	1.00	1.00	<2020
123	Nigeria	Niger	0.82	0.92	0.96	2022	1.00	1.00	1.00	<2020
128	Nigeria	Ogun	0.27	0.46	0.60	>2030	0.98	0.99	0.99	<2020
129	Nigeria	Ondo	0.38	0.63	0.81	>2030	1.00	1.00	1.00	<2020
130	Nigeria	Osun	0.35	0.63	0.82	>2030	0.99	1.00	1.00	<2020
131	Nigeria	Oyo	0.04	0.22	0.42	>2030	0.92	0.98	1.0w0	<2020
151	Nigeria	Taraba	0.17	0.25	0.31	>2030	0.67	0.73	0.76	>2030
169	Nigeria	Yobe	0.88	0.93	0.96	2022	1.00	1.00	1.00	<2020
171	Nigeria	Zamfara	0.95	0.98	0.99	<2020	1.00	1.00	1.00	<2020
69	South Sudan	East Bahr El Ghazal	0.06	0.21	0.35	>2030	0.79	0.88	0.93	2027
70	South Sudan	East Equatoria	0.11	0.20	0.31	>2030	0.72	0.83	0.88	>2030

			EPIONCHO-IBM				ONCHOSIM			
			Communtity-level results: % communities having achieved elimination by year			Year in which elimination is achieved at project level	Communtity-level results: % communities having achieved elimination by year			Year in which elimination is achieved at project level
**ID**	**Country**	**Project_name**	**2020**	**2025**	**2030**	**MDA**	**2020**	**2025**	**2030**	**MDA**
162	South Sudan	Upper Nile	0.38	0.64	0.73	>2030	0.92	0.95	0.97	<2020
163	South Sudan	West Bahr El Ghazal	0.16	0.39	0.52	>2030	0.81	0.86	0.90	>2030
164	South Sudan	West Equatoria	0.22	0.36	0.51	>2030	0.87	0.93	0.96	2022
149	Sudan	Sudan	0.90	0.99	0.99	2021	1.00	1.00	1.00	<2020
101	Tanzania	Kilosa	0.70	0.85	0.90	>2030	0.99	1.00	1.00	<2020
113	Tanzania	Mahenge	0.23	0.33	0.42	>2030	0.81	0.85	0.87	>2030
119	Tanzania	Morogoro	0.34	0.51	0.64	>2030	0.95	0.98	0.99	<2020
141	Tanzania	Ruvuma	0.14	0.19	0.24	>2030	0.59	0.65	0.69	>2030
150	Tanzania	Tanga	0.71	0.82	0.89	>2030	1.00	1.00	1.00	<2020
155	Tanzania	Tukuyu	0.53	0.61	0.66	>2030	0.95	0.97	0.98	<2020
156	Tanzania	Tunduru	0.14	0.21	0.28	>2030	0.66	0.75	0.79	>2030
136	Uganda	Phase 5	0.66	0.80	0.88	>2030	0.97	1.00	1.00	<2020

References

1. Hamley JID, Milton P, Walker M, Basáñez MG. Modelling exposure heterogeneity and density dependence in onchocerciasis using a novel individual-based transmission model, EPIONCHO-IBM: Implications for elimination and data needs. PLoS Negl Trop Dis **2019**; 13(12): e0007557.

2. Basáñez MG, Boussinesq M. Population biology of human onchocerciasis. Philos Trans R Soc Lond B Biol Sci **1999**; 354(1384): 809-26.

3. Filipe JA, Boussinesq M, Renz A, et al. Human infection patterns and heterogeneous exposure in river blindness. Proc Natl Acad Sci U S A **2005**; 102(42): 15265-70.

4. Lamberton PH, Cheke RA, Walker M, et al. Onchocerciasis transmission in Ghana: the human blood index of sibling species of the Simulium damnosum complex. Parasit Vectors **2016**; 9(1): 432.

5. Basáñez MG, Collins RC, Porter CH, Little MP, Brandling-Bennett D. Transmission intensity and the patterns of Onchocerca volvulus infection in human communities. Am J Trop Med Hyg **2002**; 67(6): 669-79.

6. Prost A. Latence parasitaire dans l'onchocercose. Bull World Health Organ **1980**; 58(6): 923-5.

7. Plaisier AP, van Oortmarssen GJ, Remme J, Habbema JD. The reproductive lifespan of *Onchocerca volvulus* in West African savanna. Acta Trop **1991**; 48(4): 271-84.

8. Duke BOL. The effects of drugs on *Onchocerca volvulus* I. Methods of assessment, population dynamics of the parasite and the effects of diethylcarbamazine. Bull World Health Organ **1968**; 39(2): 137-46.

9. Basáñez MG, Walker M, Turner HC, Coffeng LE, de Vlas SJ, Stolk WA. River Blindness: Mathematical Models for Control and Elimination. Adv Parasitol **2016**; 94: 247-341.

10. Basáñez MG, Pion SD, Boakes E, Filipe JA, Churcher TS, Boussinesq M. Effect of single-dose ivermectin on *Onchocerca volvulus*: a systematic review and meta-analysis. Lancet Infect Dis **2008**; 8(5): 310-22.

11. Eichner M. *Onchocerca volvulus* (Nematoda, Filarioidea) und *Simulium damnosum*-Komplex (Diptera): die Entwicklung intrathorakal injizierter Mikrofilarien in verschiedenen Überträgerspecies Kameruns. Germany: Universität Tübingen, **1989**.

12. Plaisier AP, Alley ES, Boatin BA, et al. Irreversible effects of ivermectin on adult parasites in onchocerciasis patients in the Onchocerciasis Control Programme in West Africa. J Infect Dis **1995**; 172(1): 204-10.

13. Dietz K. The population dynamics of onchocerciasis. In: Anderson RM. Population Dynamics of Infectious Diseases. London: Chapman and Hall, **1982**:209-41.

14. Coffeng LE, Stolk WA, Hoerauf A, et al. Elimination of African onchocerciasis: modeling the impact of increasing the frequency of ivermectin mass treatment. PLoS One **2014**; 9(12): e115886.

15. Duke BO. The population dynamics of *Onchocerca volvulus* in the human host. Trop Med Parasitol **1993**; 44(2): 61-8.

16. Plaisier AP. Modelling onchocerciasis transmission and control. Rotterdam, The Netherlands: Erasmus University, **1996**.

17. Habbema JDF, van Oortmarssen GJ, Plaisier AP. The ONCHOSIM model and its use in decision support for river blindness control. In: Isham V, Medley G. Models for infectious human diseases Their structure and relation to data. Cambridge, UK: Cambridge University Press, **1996**:360-80.

18. Duke BO. Observations on *Onchocerca volvulus* in experimentally infected chimpanzees. Tropenmed Parasitol **1980**; 31(1): 41-54.

19. Albiez EJ. Calcification in adult Onchocerca volvulus. Trop Med Parasitol **1985**; 36(3): 180-1.

20. Karam M, Schulz-Key H, Remme J. Population dynamics of Onchocerca volvulus after 7 to 8 years of vector control in West Africa. Acta Trop **1987**; 44(4): 445-57.

21. Schulz-Key H. Observations on the reproductive biology of Onchocerca volvulus. Acta Leiden **1990**; 59(1-2): 27-44.

22. Schulz-Key H, Karam M. Periodic reproduction of *Onchocerca volvulus*. Parasitol Today **1986**; 2(10): 284-6.

23. Coffeng LE, Stolk WA, Zouré HG, et al. African Programme for Onchocerciasis Control 1995-2015: model-estimated health impact and cost. PLoS Negl Trop Dis **2013**; 7(1): e2032.

24. Dadzie KY, Remme J, Rolland A, Thylefors B. The effect of 7-8 years of vector control on the evolution of ocular onchocerciasis in West African savanna. Trop Med Parasitol **1986**; 37(3): 263-70.

25. Plaisier AP, van Oortmarssen GJ, Habbema JD, Remme J, Alley ES. ONCHOSIM: a model and computer simulation program for the transmission and control of onchocerciasis. Comput Methods Programs Biomed **1990**; 31(1): 43-56.

26. Kirkwood B, Smith P, Marshall T, Prost A. Relationships between mortality, visual acuity and microfilarial load in the area of the Onchocerciasis Control Programme. Trans R Soc Trop Med Hyg **1983**; 77(6): 862-8.

27. Prost A, Vaugelade J. La surmortalité des aveugles en zone de savane ouest-africaine. Bull World Health Organ **1981**; 59: 773-6.

28. Plaisier AP, van Oortmarssen GJ, Remme J, Alley ES, Habbema JD. The risk and dynamics of onchocerciasis recrudescence after cessation of vector control. Bull World Health Organ **1991**; 69(2): 169-78.

29. Philippon B. Etude de la transmission d' *Onchocerca volvulus* (Leuckart, 1983) Nematoda, Onchocercidae) par *Simulium damnosum* (Theobald, 1903) (Diptera, Simuliidae) en Afrique tropicale. Travaux et Documents ORSTOM **1977**; 63.

30. World Health Organization. Onchocerciasis Control Programme in West Africa: report of the annual OCP research meeting. **1989**.

31. Kim YE, Remme JH, Steinmann P, Stolk WA, Roungou JB, Tediosi F. Control, elimination, and eradication of river blindness: scenarios, timelines, and ivermectin treatment needs in Africa. PLoS Negl Trop Dis **2015**; 9(4): e0003664.

32. Rebollo MP, Zouré H, Ogoussan K, Sodahlon Y, Ottesen EA, Cantey PT. Onchocerciasis: shifting the target from control to elimination requires a new first-step-elimination mapping. Int Health **2018**; 10(suppl_1): i14-i9.

33. Zarroug IM, Hashim K, ElMubark WA, et al. The first confirmed elimination of an onchocerciasis focus in Africa: Abu Hamed, Sudan. Am J Trop Med Hyg **2016**; 95(5): 1037-40.

34. Herrador Z, Garcia B, Ncogo P, et al. Interruption of onchocerciasis transmission in Bioko Island: accelerating the movement from control to elimination in Equatorial Guinea. PLoS Negl Trop Dis **2018**; 12(5): e0006471.

35. Katabarwa MN, Lakwo T, Habomugisha P, et al. After 70 years of fighting an age-old scourge, onchocerciasis in Uganda, the end is in sight. Int Health **2018**; 10(Suppl 1): i79-i88.

36. Richards FO, Eigege A, Umaru J, et al. The interruption of transmission of human onchocerciasis by an annual mass drug administration program in Plateau and Nasarawa states, Nigeria. Am J Trop Med Hyg **2020**; 102(3): 582-92.

37. Tekle AH, Elhassan E, Isiyaku S, et al. Impact of long-term treatment of onchocerciasis with ivermectin in Kaduna State, Nigeria: first evidence of the potential for elimination in the operational area of the African Programme for Onchocerciasis Control. Parasit Vectors **2012**; 5: 28.

38. Zouré HG, Noma M, Tekle AH, et al. The geographic distribution of onchocerciasis in the 20 participating countries of the African Programme for Onchocerciasis Control: (2) pre-control endemicity levels and estimated number infected. Parasit Vectors **2014**; 7: 326.

39. Coffeng LE, Pion SD, O'Hanlon S, et al. Onchocerciasis: the pre-control association between prevalence of palpable nodules and skin microfilariae. PLoS Negl Trop Dis **2013**; 7(4): e2168.
